# Identification of *LAMA1* mutations ends diagnostic odyssey and has prognostic implications for patients with presumed Joubert syndrome

**DOI:** 10.1093/braincomms/fcab163

**Published:** 2021-07-16

**Authors:** Laura Powell, Eric Olinger, Sarah Wedderburn, Vijayalakshmi Salem Ramakumaran, Usha Kini, Jill Clayton-Smith, Simon C Ramsden, Sarah J Rice, Miguel Barroso-Gil, Ian Wilson, Lorraine Cowley, Sally Johnson, Elizabeth Harris, Tara Montgomery, Marta Bertoli, Genomics England Research Consortium, Eugen Boltshauser, John A Sayer

**Affiliations:** Translational and Clinical Research Institute, Faculty of Medical Sciences, Newcastle University, Central Parkway, Newcastle upon Tyne NE1 3BZ, UK; Translational and Clinical Research Institute, Faculty of Medical Sciences, Newcastle University, Central Parkway, Newcastle upon Tyne NE1 3BZ, UK; Clinical Genetics, NHS Greater Glasgow and Clyde, Glasgow, UK; Clinical Genetics, Oxford University Hospital, Oxford, UK; Clinical Genetics, Oxford University Hospital, Oxford, UK; Manchester Centre for Genomic Medicine, Manchester University Hospitals NHS Foundation Trust, Manchester M13 9WL, UK; Division of Evolution and Genomic Sciences School of Biological Sciences, University of Manchester, Manchester M13 9PL, UK; Manchester Centre for Genomic Medicine, Manchester University Hospitals NHS Foundation Trust, Manchester M13 9WL, UK; Biosciences Institute, Faculty of Medical Sciences, Newcastle University, Central Parkway, Newcastle upon Tyne NE1 3BZ, UK; Translational and Clinical Research Institute, Faculty of Medical Sciences, Newcastle University, Central Parkway, Newcastle upon Tyne NE1 3BZ, UK; Biosciences Institute, Faculty of Medical Sciences, Newcastle University, Central Parkway, Newcastle upon Tyne NE1 3BZ, UK; Clinical Genetics, Northern Genetics Service, Newcastle upon Tyne Hospitals NHS Foundation Trust, Central Parkway, Newcastle upon Tyne NE1 3BZ, UK; Translational and Clinical Research Institute, Faculty of Medical Sciences, Newcastle University, Central Parkway, Newcastle upon Tyne NE1 3BZ, UK; Paediatric Nephrology, The Great North Children’s Hospital, Royal Victoria Infirmary, Newcastle upon Tyne NE1 4LP, UK; Clinical Genetics, Northern Genetics Service, Newcastle upon Tyne Hospitals NHS Foundation Trust, Central Parkway, Newcastle upon Tyne NE1 3BZ, UK; Clinical Genetics, Northern Genetics Service, Newcastle upon Tyne Hospitals NHS Foundation Trust, Central Parkway, Newcastle upon Tyne NE1 3BZ, UK; Clinical Genetics, Northern Genetics Service, Newcastle upon Tyne Hospitals NHS Foundation Trust, Central Parkway, Newcastle upon Tyne NE1 3BZ, UK; Paediatric Neurology (Emeritus), Children’s University Hospital, Zürich, Switzerland; Translational and Clinical Research Institute, Faculty of Medical Sciences, Newcastle University, Central Parkway, Newcastle upon Tyne NE1 3BZ, UK; Renal Services, Newcastle Upon Tyne Hospitals NHS Foundation Trust, Newcastle upon Tyne NE7 7DN, UK; NIHR Newcastle Biomedical Research Centre, Newcastle upon Tyne NE4 5PL, UK

**Keywords:** Joubert syndrome, Poretti–Boltshauser syndrome, LAMA1, cerebellar cysts, cerebellar dysplasia

## Abstract

Paediatric neurology syndromes are a broad and complex group of conditions with a large spectrum of clinical phenotypes. Joubert syndrome is a genetically heterogeneous neurological ciliopathy syndrome with molar tooth sign as the neuroimaging hallmark. We reviewed the clinical, radiological and genetic data for several families with a clinical diagnosis of Joubert syndrome but negative genetic analysis. We detected biallelic pathogenic variants in *LAMA1*, including novel alleles, in each of the four cases we report, thereby establishing a firm diagnosis of Poretti–Boltshauser syndrome. Analysis of brain MRI revealed cerebellar dysplasia and cerebellar cysts, associated with Poretti–Boltshauser syndrome and the absence of typical molar tooth signs. Using large UK patient cohorts, the relative prevalence of Joubert syndrome as a cause of intellectual disability was 0.2% and of Poretti–Boltshauser syndrome was 0.02%. We conclude that children with congenital brain disorders that mimic Joubert syndrome may have a delayed diagnosis due to poor recognition of key features on brain imaging and the lack of inclusion of *LAMA1* on molecular genetic gene panels. We advocate the inclusion of *LAMA1* genetic analysis on all intellectual disability and Joubert syndrome gene panels and promote a wider awareness of the clinical and radiological features of these syndromes.

## Teaser

Powell, Olinger et al. report four gene panel-negative kindreds with presumed Joubert syndrome where critical review of clinico-radiological data coupled with exome sequencing re-assigned a corrected diagnosis of Poretti-Boltshauser syndrome with prognostic implications. Phenotypic overlap and limited disease hallmark recognition advocate for broad, unbiased genetic testing in rare neurodevelopmental disorders.

## Graphical Abstract

**Graphical Abstract:**
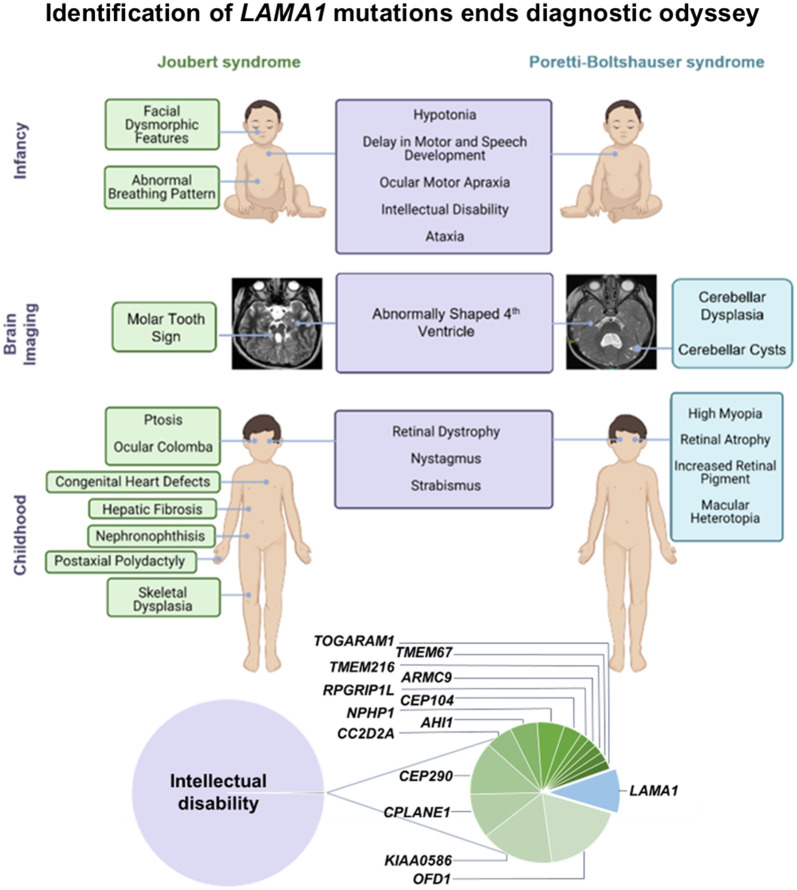

## Introduction

Poretti–Boltshauser syndrome (PTBHS) (OMIM #615960) is a rare neuro-ophthalmological disease with phenotypes that may include a non-progressive cerebellar ataxia, delayed motor and language development and intellectual disability. There are additional ophthalmological phenotypes associated with this condition that may include ocular motor apraxia (OMA), myopia, strabismus and retinal dystrophy. On brain imaging cerebellar dysplasia, cerebellar cysts and cerebellar vermis hypoplasia are typically seen. The syndrome was first described in 2014 where the features of seven children (from five unrelated families) were described.^1^ Subsequently, the underlying molecular defect was shown to be biallelic truncating or splice site mutations in *LAMA1*^2^ which encodes the Laminin alpha-1 protein. Since these initial reports of PTBHS phenotypes there has been only a handful of publications detailing additional cases.^3–7^ This suggests either that the disease remains ultra-rare, or that cases of PTBHS are not being recognized and are being incorrectly labelled. Another rare condition affecting the cerebellum is Joubert syndrome, a syndrome characterized clinically by a wide array of features including hypotonia, abnormal breathing patterns, OMA, ataxia and intellectual disability. Additional features include retinal dystrophy, nephronophthisis, liver fibrosis and skeletal dysplasia.^8^ The condition was described in 1969 by Marie Joubert^9^ and subsequently several more families with episodic hyperpnoea, abnormal eye movements and ataxia were noted.^10^ Joubert syndrome has an estimated prevalence of 1/55,000–1/200,000.^11–13^ Brain MRI in Joubert syndrome typically reveals a molar tooth sign (MTS) secondary to a deep interpeduncular fossa and elongation of the superior cerebellar peduncles.^10^ Joubert syndrome is clinically heterogeneous^13–16^ and there are currently 38 known genetic causes of Joubert syndrome.^8^

Next-generation sequencing approaches have over the past decade rapidly improved our ability to diagnose rare inherited diseases^16–20^ and these have been applied to patients with suspected Joubert syndrome.

Here, we review the clinical and imaging details of a child with developmental delay and ataxia, who had been labelled as having Joubert syndrome. We were able to establish a diagnosis of PTBHS following a review of imaging and whole-exome sequencing (WES) approaches. This prompted us to identify another three cases where similar diagnostic pitfalls led to diagnostic delays.

## Methods

### Ethical approvals and patients inclusion and clinical evaluation

This study was approved by the North East-Newcastle & North Tyneside 1 Research Ethics Committee (18/NE/350) and the Genomics England 100,000 Genomes Project was approved by the Health Research Authority Research Ethics Committee East of England—Cambridge South (REC Ref 14/EE/1112). All patients had an initial evaluation where clinical features were suggestive of Joubert syndrome with neurological phenotypes. Written and informed consent was obtained from patients and family members involved in this study.

### Genomics England 100,000 genomes project

Whole-genome sequencing (WGS) was performed through the Genomics England 100,000 Genomes Project (GE100KGP). All participants in the 100,000 Genomes Project have provided written consent to provide access to their anonymized clinical and genomic data for research purposes (https://re.extge.co.uk/ovd/) (last accessed 30/03/2021).

### Deciphering developmental disorders study

A total of 13,451 individuals with severe, undiagnosed developmental disorders were recruited from 24 regional genetics services within the United Kingdom National Health Service and the Republic of Ireland. Families gave informed consent to participate, and the study was approved by the UK Research Ethics Committee (10/H0305/83 granted by the Cambridge South Research Ethics Committee, and GEN/284/12 granted by the Republic of Ireland Research Ethics Committee). Details on sample collection and genetic analysis pipelines have been described previously.^21^ Genetic variants and linked phenotypic data have been accessed via DECIPHER (https://www.deciphergenomics.org/) (last access 30/03/21).

### Variants validation by Sanger sequencing

Sanger sequencing was utilized to confirm variants and their segregation from both parents where DNA samples where available. PCR amplification was performed using *Taq* PCR master mix (Qiagen) kit, as per the manufacturer’s instructions.

### Data availability statement

The authors confirm that the data supporting the findings of this study are available within the article and its supplementary materials. Further phenotypic or sequencing data are available from the corresponding author (J.A.S.), upon reasonable request. Genetic variants and linked phenotypic data from the deciphering developmental disorders study can be accessed via DECIPHER (https://www.deciphergenomics.org/). The novel genetic variants in *LAMA1* have been submitted to ClinVar.

## Results

### Patients characteristics

**Figure 1:**
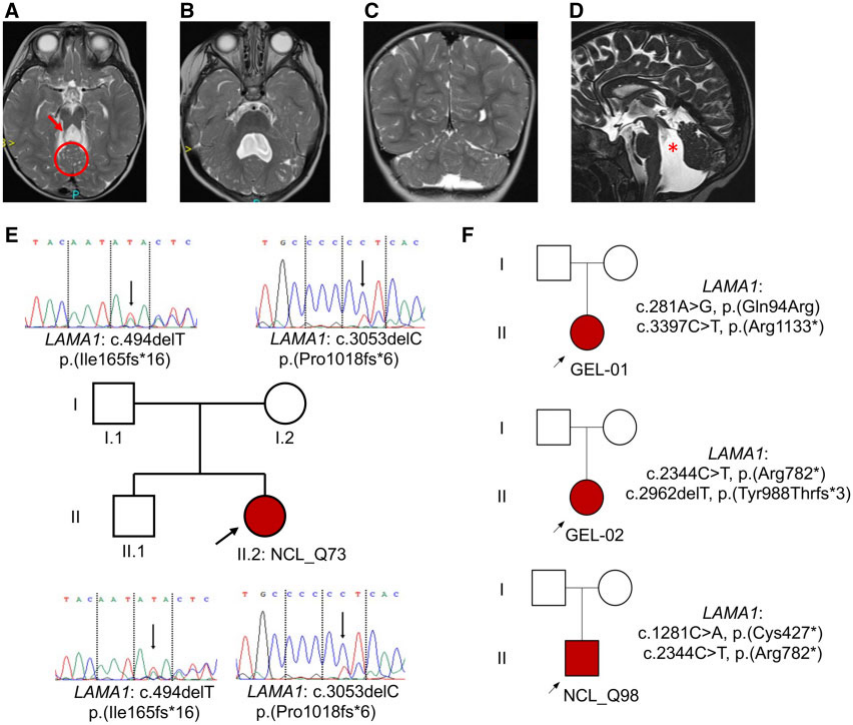
Diagnosis of Poretti–Boltshauser syndrome in four British families. Brain MRI findings in proband NCL_Q73 demonstrate (**A**) axial view at the level of the upper vermis showing multiple cysts (circle) and elongated splayed superior cerebellar peduncles (arrow); (**B**) axial view demonstrating an abnormal shaped enlarged fourth ventricle and multiple cysts in the peripheral parts of the hemispheres; (**C**) coronal view showing multiple cysts in the upper parts of the cerebellum and marked dysplasia; (**D**) sagittal view demonstrating an enlarged quadrangular fourth ventricle (asterisk) and small cysts in the anterior vermis.(**E**) *LAMA1* molecular genetic results in index case NCL_Q73 and (**F**) three additional cases identified in this study. Probands are identified by arrows. Shaded symbols show affected cases, males squares and females circles.

The index family proband (NCL_Q73) was a child of 3 years of age that had been labelled as Joubert syndrome given her clinical features of ataxia, OMA and a possible molar tooth sign detected on brain MRI imaging (Table 1, Fig. 1). A previous genetic analysis using a next-generation sequencing 29 genes panel of Joubert syndrome genes was negative. Her history was reviewed as well as her brain imaging (Table 1) and features seemed to diverge from a typical Joubert syndrome presentation. In particular, the brain MRI scans showed evidence of cerebellar cysts (Fig. 1, Supplementary Tables 1 and 2, Supplementary Figs 1–4).

**Table 1:** Clinical and neuro-imaging features of patients

Patient ID	NCL_Q73	GEL-01	GEL-02	NCL_Q98
Age	3 years	6 years 11 months	8 years	12 years
Sex	F	F	F	M
Ethnicity	White British	Caribbean/Gambian	White British	White British
Neurodevelopmental features	Moderate speech delay	Mild motor and speech delay	Motor delay	Motor and speech delay
Ataxia	Ataxia	Truncal and gait ataxia	Truncal Ataxia	Ataxia
Autistic features	Yes	No	No	Yes
Ocular motor apraxia	Yes	No	No	Yes
Myopia	Yes	Yes	Yes	Yes
Cerebellar dysplasia	Yes	Yes	Yes	Yes
Cerebellar cysts	Yes	Yes	Yes	Yes
Molar tooth sign	No	No	No	No
Additional brain MRI features	Splayed superior cerebellar peduncles	Abnormal shape of the fourth ventricle	Abnormal rhomboid shape of fourth ventricle in axial and sagittal plane, Splayed superior cerebellar peduncles	Abnormal rhomboid shape of fourth ventricle in axial and sagittal plane
Other		Total anomalous pulmonary venous drainage	Branchial cyst	

### Exome sequencing data

Following informed consent, duo WES was carried out for the proband and her mother in order to pursue a precise molecular genetic diagnosis. WES data was initially filtered for biallelic changes in 38 genes known to cause Joubert syndrome (Supplementary Table 3) and monoallelic changes in *SUFU*. This led to no obvious underlying genetic causes in this set of genes. Filtering of variants was adjusted to examine all rare variants within the exome dataset and this identified two rare and predicted pathogenic alleles in *LAMA1* (Fig. 1, Table 2). Using DNA obtained from both parents and the proband we confirmed the variants in *LAMA1* by Sanger sequencing as well as their segregation from each parent. Biallelic variants in *LAMA1* cause PTBHS, which would fully account for the clinical presentation and phenotype. The identification of this index case of PTBHS and the diagnostic pitfalls leading to an erroneous clinical diagnosis of Joubert syndrome led us to search for other similar mislabelled cases.

**Table 2:** Molecular genetic investigations

	Patient ID	Prior molecular genetic tests	Molecular tests applied in this study	*LAMA1* alleles identified (NM_005559.4)	Pathogenicity (MutationTaster) & gnomAD allele frequency (Allele counts/Homoz/Total alleles)
	NCL_Q73	29 genes panel for Joubert syndrome: negative	Duo WES (proband and mother)	c.494delT, p.(Ile165fs*16) (Het); c.3053delC, p.(Pro1018fs*6) (Het)	Disease causing; not in gnomAD Disease causing; not in gnomAD
	GEL-01	WGS (Genomics England) virtual panel: unsolved	Re-evaluation of WGS data	c.281A>G, p.(Gln94Arg)^a^ (Het); 2. c.3397C>T, p.(Arg1133*) (Het)	Disease causing; not in gnomAD Disease causing; 5/0/250388
	GEL-02	Hereditary ataxia gene panel: negative, WGS (Genomics England) virtual panel: unsolved	Re-evaluation of WGS data	c.2344C>T, p.(Arg782*) (Het); c.2962delT, p.(Tyr988Thrfs*3) (Het)	Disease causing, ClinVar: Pathogenic; 11/0/272248 Disease causing; not in gnomAD
	NCL_Q98	18 genes panel for Joubert syndrome: negative Microarray analysis Mat inherited variant 4q28.1, likely benign	DDD study recruitment Array CGH and WES	c.1281C>A, p.(Cys427*) (Het); c.2344C>T, p.(Arg782*) (Het)	Disease causing; not in gnomAD Disease causing, ClinVar: Pathogenic; 11/0/272248

a MutationTaster: ‘Disease Causing’; SIFT: ‘deleterious’; PolyPhen: ‘probably damaging’; CADD score 26.4; REVEL score 0.923. Gln94 is highly conserved and lies within the Laminin alpha-1 LN domain (Supplementary Fig. 6).

CGH = comparative genomic hybridization; gnomAD = genome aggregation database; het = heterozygous; homoz = homozygous; WES = whole exome sequencing; WGS = whole-genome sequencing.

### Identification of additional patients with undiagnosed PTBHS

The 100,000 Genomes Project provides a rich source of WGS data on patients with rare disease phenotypes.^22^ We searched the whole rare disease dataset (73,988 genomes) for pathogenic alleles in *LAMA1* and identified 2 probands (GEL-01 and GEL-02) with rare, pathogenic biallelic changes in *LAMA1* (Fig. 1, Tables 1 and 2, Supplementary Figs 5 and 6) and brain MRI phenotypes consistent with PTBHS (Supplementary Figs 7 and 8). These alleles had been filtered out by the standard tiering tables and the patients’ physicians had received an ‘exit questionnaire’ suggesting no causative mutations had been identified and that the patients remained genetically unsolved. The most likely reason for this is that these patients were recruited into phenotypic groups ‘Congenital malformations caused by ciliopathies’ (specific disease Joubert syndrome) for patient GEL-01 and ‘Motor disorders of the CNS’ (specific disease cerebellar hypoplasia) for patient GEL-02, neither of which contained *LAMA1* as a causative gene in the virtual gene panel applied. We have subsequently added *LAMA1* as a potential causative gene to these panels via the Genomics England PanelApp (https://panelapp.genomicsengland.co.uk) so that future cases will not be missed. Finally, we examined the DECIPHER database^23^ (https://decipher.sanger.ac.uk/) for patients with *LAMA1* mutations and found an additional UK family with a proband (NCL_Q98) who had been clinically labelled Joubert syndrome at the age of 14  months based on the presence of OMA and an abnormal MRI brain scan (Fig. 1, Tables 1 and 2, Supplementary Fig. 9). A gene panel for Joubert syndrome had been previously performed which showed no mutations. The family was enrolled in the deciphering developmental disorders study which had revealed a molecular genetic diagnosis of *LAMA1* mutations (Supplementary Fig. 5) and a clinical diagnosis was confirmed to be PTBHS.^24^

Finally, we examined published cases of PTBHS in the literature to determine longer-term outcomes for patients with this condition, given that this is a frequently asked question by both physicians and affected family members (Supplementary Table 4). Data are limited but there is some evidence of extremely good outcomes including adult patients with normal IQ levels, attending college and higher education and running their own businesses. The caveat is that the majority of known patients with PTBHS are still children and the long-term outlook is not yet known.

### Estimated prevalence of PTBHS and Joubert syndrome in UK patient cohorts

**Figure 2:**
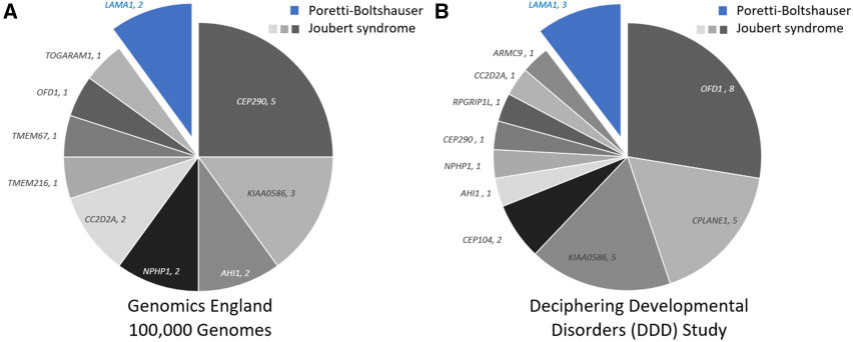
Genetic diagnosis of Poretti–Boltshauser syndrome and Joubert syndrome in patients with developmental disorders and intellectual disabilities. (**A**) Number of cases solved for either *LAMA1* (blue) or Joubert syndrome genes (greyscale) among 8459 probands recruited with human phenotype ontology terms ‘intellectual disability’ and/or ‘developmental delay’ in Genomics England 100 000 Genomes Project. (**B**) Number of cases solved for either *LAMA1* (blue) or Joubert syndrome genes (greyscale) among ∼14 000 probands recruited in the UK Deciphering Developmental Disorders study and with phenotypical data indicating either intellectual disability or developmental delay.

In order to assess the relative prevalence of molecular diagnoses of PTBHS and Joubert syndrome in patients with intellectual disabilities and developmental disorders, we searched the Genomics England (GEL) 100,000 Genomes Project rare disease dataset as well as deciphering developmental disorders study for cases solved with either one of the 38 known Joubert syndrome genes (Supplementary Table 3) or pathogenic variants in *LAMA1*. Out of 8459 probands recruited in GEL with human phenotype ontology terms ‘intellectual disability’ and/or ‘developmental delay’, 18 cases (0.2%) were molecularly solved for Joubert syndrome genes versus only two cases solved with *LAMA1* variants (Fig. 2A). Similarly, among ∼14,000 individuals with developmental disorders recruited in the UK deciphering developmental disorders study, 26 (∼0.2%) were solved for a Joubert syndrome gene and three cases solved with *LAMA1* variants (Fig. 2B). Molecular genetics approaches, unbiased for clinical or radiological assessments, indicate thus that PTBHS is a considerably rarer cause of developmental disorders and intellectual disability than the collective of genes causing Joubert syndrome, at least in these UK cohorts. On the other hand, the relative contribution of *LAMA1* to developmental disorders is likely comparable to the more common among the Joubert syndrome genes, when considered individually (Fig. 2).

## Discussion

**Figure 3:**
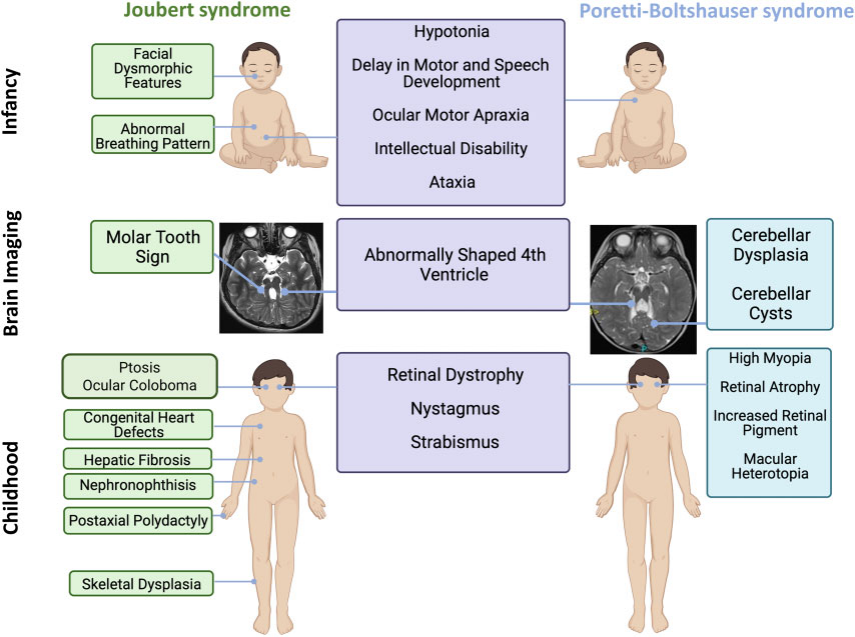
Infographic comparing Poretti–Boltshauser syndrome and Joubert syndrome. A visual comparison of Joubert syndrome and Poretti–Boltshauser syndrome during infancy, with axial brain imaging and during childhood highlights their shared and distinguishing features.

Joubert syndrome and PTBHS can present in very similar ways in *infancy,* and this may cause diagnostic uncertainty and confusion (Supplementary Table 1, Fig. 3). Clearly, it is vital that at this time, when patients are ‘deviating’ from normal development, appropriate investigations are planned. For multisystem syndromes such as Joubert syndrome it is desirable for an *early* diagnosis to be made, for example before the onset of end-stage kidney disease. For a more limited condition, there may be less urgency, but both family members and physicians will appreciate a precise diagnostic label so appropriate management plans can be put in place and the diagnostic work-up stopped. This is also relevant when considering allocations of public healthcare resources, as a molecular diagnosis of PTBHS implies that extra-CNS manifestations are unlikely to occur and do not need to be regularly screened for, in contrast to Joubert syndrome.^8^ The main clinical similarities between Joubert syndrome and PTBHS are delays in motor and speech development, and OMA in the vast majority (Supplementary Table 1). A recent observation is that autosomal dominant variant in *SUFU* cause a similar clinical phenotype in infancy.^25^ We emphasize that the clinical findings in infancy do overlap but later in childhood the syndromes of Joubert syndrome and PTBHS diverge.

Regarding brain imaging, pattern recognition is required for the identification and distinction of the two syndromes. The radiologist needs high-definition images in order to make a correct diagnosis and needs to be qualified in the interpretation of neurological conditions. The field of posterior fossa anomalies may not be an area of expertise for most radiologists. This point is important as the correct interpretation of brain imaging will lead to the correct set of genetic investigations to be performed and will result in a faster, more cost-effective diagnostic odyssey for the patient.

 For Joubert syndrome, the MRI imaging hallmark is the molar tooth sign (MTS), but in practice, there is an imaging spectrum of additional infra- and supratentorial anomalies.^26^ It is noteworthy that the MTS is variable in peculiarity—from very pronounced to less marked, in the literature also called ‘mild MTS’. Non-specialists may easily overlook mild MTS features on brain MRI. The findings of mild MTS have often been reported in Joubert syndrome associated with mutations in *NPHP1*, *C5orf42*, *FAM149B1*, *CBY1.*^27^ The mild MTS seen in some types of Joubert syndrome is not distinguishable from mild MTS in patients with *SUFU* variants, emphasizing the role of targeted molecular genetic investigations for these conditions. The differential diagnosis for MTS includes pontine cap dysplasia, but the clinical context and remainder of imaging are very different.^28^

We would like to emphasize that there is no imaging overlap between Joubert syndrome and PTBHS (Supplementary Table 2). In PTBHS brain imaging there is a ‘hierarchy’ of findings, of which cerebellar dysplasia is the leading sign, followed by cerebellar cysts. The differential diagnosis of cerebellar dysplasia and cysts includes some severe forms of congenital muscular dystrophies however, other clinical contexts and additional MRI anomalies do not allow for diagnostic confusion. The presence of a typical MTS has a strong impact on the presumed diagnosis and the further workup. Given these implications, the clinician that requested MRI images should carefully review the imaging findings and act as a ‘gate-keeper’ for a premature label of MTS and Joubert syndrome.

The accessibility of WGS and WES in both research and clinical care settings has allowed huge advances in our understanding and diagnosis of rare diseases.^22^^,^^29^ Indeed, genetic work-up has become standard of care for several neurodevelopmental disorders and the diagnostic yields by WES in patients with an intellectual disability or global developmental delay is estimated around 35%.^30^ The testing modalities (sequencing, array-based techniques, karyotyping) and scope of genetic testing (selected gene panel, WES or WGS) depend on the clinical presentation and recommendations are likely to evolve with technological development. Emerging evidence suggests that WES may be preferable over a targeted analysis of selected genes and that WES also outperforms chromosomal microarrays as an initial testing approach in neurodevelopmental disease.^31^ Although overall coverage is usually better with targeted analyses, WES was shown to cover >98% of mutations identified on targeted next-generation sequencing panels.^32^ Interestingly, a simulation study that asked clinicians to choose a commercially available gene panel as an alternative when ordering singleton WES for the various suspected monogenic diseases found that 23% of patients would not have been solved had this panel been applied instead of WES.^33^ What is more, panel analyses are quickly outdated as novel genetic aetiologies for disease emerge and gene discovery in the arena of neurodevelopmental disorders is fast moving.^20^^,^^27^ As an example, 11% of patients negative on a comprehensive ‘Mendeliome’ panel received a diagnosis via WES, simply because the panel did not include the most recent gene–disease relationships identified after the panel was established.^34^ Finally, WES is cost-efficient, with a considerable decrease in healthcare expenditures and downstream medical interventions once WES was performed and WES also compared favourably with targeted analysis, especially if a negative panel analysis leads to further investigations and subsequent re-analysis by WES or WGS.^33^^,^^35^ Indeed, the cases that we report here corroborate the notion that negative findings in a selected gene panel analysis should be followed-up by an unbiased investigation using WES or WGS. Taken together, the limitations of panel analyses are not of technical nature; on the contrary, targeted analyses usually provide increased read depth, sequence coverage and detection of exon-level deletions and/or duplications. The examples above as well as the cases reported here rather highlight the difficulties for an *a priori* assignment of potential genetic causes to a phenotype. This is an important limitation for gene panel analysis, where precise phenotypic characterization is required and dictates the set of genes that are investigated. Virtual gene panels, where untargeted WES or WGS is performed, and variant interpretation then restricted to a selected number of genes have several advantages: generated data is easier to manage, secondary findings are unlikely to occur, but the scope of analysis can be extended if no pathogenic variants are detected, or new genes are shown to cause the investigated phenotype. We have shown here that there is a certain phenotypic overlap, especially in infancy, between Joubert syndrome and PTBHS, and therefore it seems a pragmatic approach to add the single *LAMA1* gene to Joubert syndrome gene panels so this alternate diagnosis can be detected. In addition, efforts to re-analyse unsolved Joubert syndrome-like patients and those with cerebellar dysplasia and cysts should be made by opening up virtual gene panels to other genetic phenocopies, such as *LAMA1*. Expert multidisciplinary teams can be utilized to update and extend virtual gene panels and report back the alleles and their pathogenicity in context with the clinical phenotype, which can evolve as the child develops.

In discovering these cases of PTBHS, we were struck by the profound impact the genetic diagnosis made on each of the families. In all four cases, there was a delay caused by incomplete genetic analysis and failure to include *LAMA1* in the gene panel applied. Due to this lack of molecular genetic diagnosis, in all four cases, the clinical diagnosis defaulted to Joubert syndrome. The molecular genetic diagnosis of *LAMA1* mutations led to a new diagnostic label and a feeling of loss and insecurity for the families involved. A new diagnosis also has some prognostic implications. Compared to the multisystem features of Joubert syndrome, PTBHS has a much more limited phenotype. To date, no other organs aside from the brain are involved and there is no evidence for disease progression. In particular, it is not associated with the renal and liver problems which may be seen in Joubert syndrome and individuals with an established diagnosis of PTBHS do not require regular monitoring for these. Although OMA persists into adult life. With that in mind, we tried to draw some conclusions from the older patients that have been reported in the literature. This confirmed the non-progressive nature of the PTBHS phenotype and overall favourable outcomes including some examples of normal intellectual ability and independence but we suspect this data is both incomplete and subject to bias. In line with a somewhat milder phenotype, we also detected a ∼50-year-old individual in gnomAD carrying a homozygous predicted loss-of-function variant in *LAMA1* (c.858+1G>T).

Next-generation sequencing allows an unbiased analysis of the whole exome or genome and has become more widely available for use as a first-line diagnostic tool for the investigation of patients with neurodevelopmental disease, including cerebellar disorders. It has the potential to resolve those cases with suspected Joubert syndrome and disorders such as PTBHS which may clinically mimic Joubert syndrome in infancy.^36^ We present four real-world examples highlighting the strength of an unbiased genetic approach over a targeted panel analysis and of how a precise molecular diagnosis allows some clarity in terms of clinical monitoring for the physician and potential long-term outcomes for the patient.

## Supplementary material

Supplementary material is available at *Brain Communications* online.

## Supplementary Material

fcab163_Supplementary_Data

## Appendix

### Genomics England Research Consortium

Ambrose, J. C.; Arumugam, P.; Bleda, M.; Boardman-Pretty, F.; Boustred, C. R.; Brittain, H; Caulfield, M. J.; Chan, G. C.; Fowler, T.; Giess A.; Hamblin, A.; Henderson, S.; Hubbard, T. J. P.; Jackson, R.; Jones, L. J.; Kasperaviciute, D.; Kayikci, M.; Kousathanas, A.; Lahnstein, L.; Leigh, S. E. A.; Leong, I. U. S.; Lopez, F. J.; Maleady-Crowe, F.; Moutsianas, L.; Mueller, M.; Murugaesu, N.; Need, A. C.; O‘Donovan P.; Odhams, C. A.; Patch, C.; Perez-Gil, D.; Pereira, M. B.; Pullinger, J.; Rahim, T.; Rendon, A.; Rogers, T.; Savage, K.; Sawant, K.; Scott, R. H.; Siddiq, A.; Sieghart, A.; Smith, S. C.; Sosinsky, A.; Stuckey, A.; Tanguy M.; Thomas, E. R. A.; Thompson, S. R.; Tucci, A.; Walsh, E.; Welland, M. J.; Williams, E.; Witkowska, K.; Wood, S. M.

**Abbreviated summary;** Powell, Olinger et al. report four gene panel-negative kindreds with presumed Joubert syndrome where critical review of clinico-radiological data coupled with exome or genome sequencing re-assigned a corrected diagnosis of Poretti–Boltshauser syndrome with prognostic implications. Phenotypic overlap and limited disease hallmark recognition advocate for broad, unbiased genetic testing in rare neurodevelopmental disorders.

## Abbreviations

DDD =: deciphering developmental disorders
GEL =: Genomics England
MTS =: Molar tooth sign
OMA =: ocular motor apraxia
PTBHS =: Poretti–Boltshauser syndrome
WES =: Whole-exome sequencing
