# Supplementary material for: Identification of LAMA1 mutations ends diagnostic odyssey and has prognostic implications for patients with presumed Joubert syndrome
Source: Brain Commun. 2021 Jul 16;3(3):fcab163. doi: 10.1093/braincomms/fcab163 (PMC8374969; doi:10.1093/braincomms/fcab163)
Supplement: fcab163_Supplementary_Data [file fcab163_Supplementary_Data.zip › Supplementary_material_2.docx]

Supplementary Information

**Identification of *LAMA1* mutations ends diagnostic odyssey and has prognostic implications for patients with presumed Joubert syndrome**

Appendix: Genomics England Research Consortium

Supplementary Tables 1-4

Supplementary Figures 1-9

Supplementary References

**Appendix: Genomics England Research Consortium**

Ambrose, J. C. ^1^ ; Arumugam, P.^1^ ; Bleda, M. ^1^ ; Boardman-Pretty, F. ^1,2^ ; Boustred, C. R. ^1^ ; Brittain, H.^1^ ; Caulfield, M. J.^1,2^ ; Chan, G. C. ^1^ ; Fowler, T. ^1^ ; Giess A. ^1^ ; Hamblin, A.^1^ ; Henderson, S.^1,2^ ; Hubbard, T. J. P. ^1^ ; Jackson, R. ^1^ ; Jones, L. J. ^1,2^ ; Kasperaviciute, D. ^1,2^ ; Kayikci, M. ^1^ ; Kousathanas, A. ^1^ ; Lahnstein, L. ^1^ ; Leigh, S. E. A. ^1^ ; Leong, I. U. S. ^1^ ; Lopez, F. J. ^1^ ; Maleady-Crowe, F. ^1^ ; Moutsianas, L. ^1,2^ ; Mueller, M. ^1,2^ ; Murugaesu, N. ^1^ ; Need, A. C. ^1,2^ ; O‘Donovan P. ^1^ ; Odhams, C. A. ^1^ ; Patch, C. ^1,2^ ; Perez-Gil, D. ^1^ ; Pereira, M. B. ^1^ ; Pullinger, J. ^1^ ; Rahim, T. ^1^ ; Rendon, A. ^1^ ; Rogers, T. ^1^ ; Savage, K. ^1^ ; Sawant, K. ^1^ ; Scott, R. H. ^1^ ; Siddiq, A. ^1^ ; Sieghart, A. ^1^ ; Smith, S. C. ^1^ ; Sosinsky, A. ^1,2^ ; Stuckey, A. ^1^ ; Tanguy M. ^1^ ; Thomas, E. R. A. ^1,2^ ; Thompson, S. R. ^1^ ; Tucci, A. ^1,2^ ; Walsh, E. ^1^ ; Welland, M. J. ^1^ ; Williams, E. ^1^ ; Witkowska, K. ^1,2^ ; Wood, S. M. ^1,2^.

^1^ Genomics England, London, UK

^2^ William Harvey Research Institute, Queen Mary University of London, London, EC1M 6BQ, UK

**Supplementary Table 1. Comparison of clinical features of Joubert syndrome and Poretti-Boltshauser syndrome**

|  | **Joubert Syndrome** | **Poretti-Boltshauser Syndrome** |
| --- | --- | --- |
| **Infancy** | Hypotonia  Delay in motor and speech development  Ocular motor apraxia  Intellectual disability  Ataxia  Abnormal breathing pattern (not consistent)  Facial dysmorphic features, including frontal bossing, high-arched eyebrows, epicanthic folds, anteverted nostrils, long philtrum, open mouth, protruded tongue (not consistent) | Hypotonia  Delay in motor and speech development  Ocular motor apraxia  Intellectual disability  Ataxia |
| **Additional eye phenotypes** | Retinal dystrophy  Nystagmus  Strabismus  Ptosis  Ocular coloboma | Retinal dystrophy  Nystagmus  Strabismus  Retinal atrophy  Increased retinal pigment  Macular heterotopia  High myopia |
| **Other phenotypes** | Nephronophthisis  Hepatic fibrosis  Postaxial polydactyly  Bone shortening, skeletal dysplasia  Oral hamartomas  Congenital heart defects |  |

Red are shared features, black may be distinguishing features, but are not consistent or age dependent

**Supplementary Table 2. Comparison of brain MRI features of Joubert syndrome and Poretti-Boltshauser syndrome (see Figures S1-S4)**

| **Joubert Syndrome** | **Poretti-Boltshauser Syndrome** |
| --- | --- |
| Molar Tooth Sign (resulting from deep interpeduncular fossa and elongated thickened superior cerebellar peduncles)  Cerebellar vermis hypoplasia / dysplasia  Upper vermis: folial dysplasia  Abnormal shape 4^th^ ventricle, dislocated fastigium  (No cerebellar cysts) | Cerebellar dysplasia  Cerebellar cysts  Abnormally shaped 4^th^ ventricle (in axial and sagittal plane)  Superior cerebellar peduncles splayed (coronal and axial) |

**Supplementary Table 3. Known genetic causes of Joubert syndrome**

| Phenotype | Gene | Gene MIM number |
| --- | --- | --- |
| [Joubert syndrome 1](https://omim.org/entry/213300) | [*INPP5E*](https://omim.org/entry/613037) | [613037](https://omim.org/entry/613037) |
| [Joubert syndrome 2](https://omim.org/entry/608091) | [*TMEM216*](https://omim.org/entry/613277) | [613277](https://omim.org/entry/613277) |
| [Joubert syndrome 3](https://omim.org/entry/608629) | [*AHI1*](https://omim.org/entry/608894) | [608894](https://omim.org/entry/608894) |
| [Joubert syndrome 4](https://omim.org/entry/609583) | [*NPHP1*](https://omim.org/entry/607100) | [607100](https://omim.org/entry/607100) |
| [Joubert syndrome 5](https://omim.org/entry/610188) | [*CEP290*](https://omim.org/entry/610142) | [610142](https://omim.org/entry/610142) |
| [Joubert syndrome 6](https://omim.org/entry/610688) | [*TMEM67*](https://omim.org/entry/609884) | [609884](https://omim.org/entry/609884) |
| [Joubert syndrome 7](https://omim.org/entry/611560) | [*RPGRIP1L*](https://omim.org/entry/610937) | [610937](https://omim.org/entry/610937) |
| [Joubert syndrome 8](https://omim.org/entry/612291) | [*ARL13B*](https://omim.org/entry/608922) | [608922](https://omim.org/entry/608922) |
| [Joubert syndrome 9](https://omim.org/entry/612285) | [*CC2D2A*](https://omim.org/entry/612013) | [612013](https://omim.org/entry/612013) |
| [Joubert syndrome 10](https://omim.org/entry/300804) | [*OFD1*](https://omim.org/entry/300170) | [300170](https://omim.org/entry/300170) |
| Joubert syndrome 11 | *TTC21B* | 612014 |
| [Joubert syndrome 12](https://omim.org/entry/200990) | [*KIF7*](https://omim.org/entry/611254) | [611254](https://omim.org/entry/611254) |
| [Joubert syndrome 13](https://omim.org/entry/614173) | [*TECT1*](https://omim.org/entry/609863)*/TCTN1* | [609863](https://omim.org/entry/609863) |
| [Joubert syndrome 14](https://omim.org/entry/614424) | [*TMEM237*](https://omim.org/entry/614423) | [614423](https://omim.org/entry/614423) |
| [Joubert syndrome 15](https://omim.org/entry/614464) | [*CEP41*](https://omim.org/entry/610523) | [610523](https://omim.org/entry/610523) |
| [Joubert syndrome 16](https://omim.org/entry/614465) | [*TMEM138*](https://omim.org/entry/614459) | [614459](https://omim.org/entry/614459) |
| [Joubert syndrome 17](https://omim.org/entry/614615) | [*CPLANE1*](https://omim.org/entry/614571)*/C5orf42* | [614571](https://omim.org/entry/614571) |
| [Joubert syndrome 18](https://omim.org/entry/614815) | [*TCTN3*](https://omim.org/entry/613847) | [613847](https://omim.org/entry/613847) |
| [Joubert syndrome 19](https://omim.org/entry/614844) | [*ZNF423*](https://omim.org/entry/604557) | [604557](https://omim.org/entry/604557) |
| [Joubert syndrome 20](https://omim.org/entry/614970) | [*TMEM231*](https://omim.org/entry/614949) | [614949](https://omim.org/entry/614949) |
| [Joubert syndrome 21](https://omim.org/entry/615636) | [*CSPP1*](https://omim.org/entry/611654) | [611654](https://omim.org/entry/611654) |
| [Joubert syndrome 22](https://omim.org/entry/615665) | [*PDE6D*](https://omim.org/entry/602676) | [602676](https://omim.org/entry/602676) |
| [Joubert syndrome 23](https://omim.org/entry/616490) | [*KIAA0586*](https://omim.org/entry/610178) | [610178](https://omim.org/entry/610178) |
| [Joubert syndrome 24](https://omim.org/entry/616654) | [*TCTN2*](https://omim.org/entry/613846) | [613846](https://omim.org/entry/613846) |
| [Joubert syndrome 25](https://omim.org/entry/616781) | [*CEP104*](https://omim.org/entry/616690) | [616690](https://omim.org/entry/616690) |
| [Joubert syndrome 26](https://omim.org/entry/616784) | [*KATNIP*](https://omim.org/entry/616650) | [616650](https://omim.org/entry/616650) |
| [Joubert syndrome 27](https://omim.org/entry/617120) | [*B9D1*](https://omim.org/entry/614144) | [614144](https://omim.org/entry/614144) |
| [Joubert syndrome 28](https://omim.org/entry/617121) | [*MKS1*](https://omim.org/entry/609883) | [609883](https://omim.org/entry/609883) |
| [?Joubert syndrome 29](https://omim.org/entry/617562) | [*TMEM107*](https://omim.org/entry/616183) | [616183](https://omim.org/entry/616183) |
| [Joubert syndrome 30](https://omim.org/entry/617622) | [*ARMC9*](https://omim.org/entry/617612) | [617612](https://omim.org/entry/617612) |
| [Joubert syndrome 31](https://omim.org/entry/617761) | [*CEP120*](https://omim.org/entry/613446) | [613446](https://omim.org/entry/613446) |
| [Joubert syndrome 32](https://omim.org/entry/617757) | [*SUFU*](https://omim.org/entry/607035) | [607035](https://omim.org/entry/607035) |
| [Joubert syndrome 33](https://omim.org/entry/617767) | [*PIBF1*](https://omim.org/entry/607532) | [607532](https://omim.org/entry/607532) |
| Joubert syndrome 34 | *B9D2* | 614175 |
| [Joubert syndrome 35](https://omim.org/entry/618161) | [*ARL3*](https://omim.org/entry/604695) | [604695](https://omim.org/entry/604695) |
| [Joubert syndrome 36](https://omim.org/entry/618763) | [*FAM149B1*](https://omim.org/entry/618413) | 618413 |
| Joubert syndrome 37 | *TOGARAM1* | 617618 |
| N/A | *CBY1* | N/A^1^ |

**Supplementary Table 4. Summary of reported Poretti-Boltshauser patients**

| **Patient ID from Publication** | ***LAMA1* variants**  **(NM_005559)** | **Age of MRI / Diagnosis** | **Educational Achievement (age)** | **REF** |
| --- | --- | --- | --- | --- |
| CA0035 | c.588+2T>G (Hom) | 36 months at diagnosis | Not known (36 m) | ^2^ |
| UW154-3 | c.6345+3G>C (Het);  Del exons 4-11 (Het) | 36 months at diagnosis | Not known (36 m) | ^2^ |
| UW162-3 | c.7965-15_79653del (Het);  c.2988_2989delA, p.(Pro996Hisfs28*) (Het) | 9 months at diagnosis | Not known (25 months) | ^2^ |
| UW160-3 | c.6701delC, p.(Pro2334Leufs9*) (Het);  c.8557-1G>C (Het);  c.768+1G>A (Het) | Unknown | UW 160-3 at 29 years - Normal IQ  College graduate, lives independently | ^2^ |
| UW160-4 | c.6701delC, p.(Pro2334Leufs9*) (Het);  c.8557-1G>C (Het);  c.768+1G>A (Het) | Unknown | UW 160-4 At 23 years autism spectrum disorder (Asperger), lives with parents | ^2^ |
| UW163-3  (Family 1, Patient 1) | c.2816_2817delAT, p.(Tyr939Leu27*) (Het);  c.555T>G, p.(Tyr185*) (Het) | 2 years 1 month | Not known (26 years) | ^2,3^ |
| UW163-4  (Family 1, Patient 2) | c.2816_2817delAT, p.(Tyr939Leu27*) (Het);  c.555T>G, p.(Tyr185*) (Het) | 5 months | Not known (21 years) | ^2,3^ |
| Family 2, Patient 3 | c.2160T>A, p.(Cys720*) (Het);  c.5985_5991del, p.(Ile1996Glufs*7) (Het) | 8 years 6 months | Not known (8 years 6 months) | ^3^ |
| Patient 1 | c.664C>T, p.(Arg222*) (Het);  c.2331C>G, p.(Tyr777*) (Het) | <5 years | Not known (8 years) | ^4^ |
| Patient 2 | c.664C>T, p.(Arg222*) (Het);  c.2331C>G, p.(Tyr777*) (Het) | <5 years | Not known (8 years) | ^4^ |
| Patient 1 | c.4702_4703del; p.(Leu1568Glyfs*2) (Hom) | 2 years 6 months | Not known (2 years 6 months) | ^5^ |
| Patient 1 | c.8192C>A, p.(Ser2731*) (Hom) | 7 years | Not known (7 years) | ^6^ |
| Patient 2 | c.8192C>A, p.(Ser2731*) (Hom) | 7 years | Not known (7 years( | ^6^ |
| Patient 1 | c. 8556+1G>A (Hom) | 4 months | IQ 70 (12 years 4 months) | ^7^ |
| Patient 2 | c.2935delA, p.(Arg979Glyfs*45) (Hom) | 5 months | IQ 60 (7 years 4 months) | ^7^ |
| Patient 3 | c.4676delA, p.(Glu1559Glyfs*3) (Het)  c.7180C>T; p.(Arg2394*) (Het) | 3 months | Developmental delay (6 years 6 months) | ^7^ |
| Patient 4 | c.2935delA, p.(Arg979Glyfs*45) (Hom) | 3-6 months | Developmental delay (14 years) | ^7^ |
| Patient 5 | c.2935delA, p.(Arg979Glyfs*45) (Hom) | 3-6 months | Developmental delay (8 years 9 months) | ^7^ |
| Patient 6 | c.2935delA, p.(Arg979Glyfs*45) (Hom) | 3-6 months | Developmental delay (7 years 6 months) | ^7^ |
| Patient 7 | c.2935delA, p.(Arg979Glyfs*45) (Hom) | 3-6 months | Developmental delay (11 years) | ^7^ |
| Patient 8 | c.1774_1775insTTCATAAT, p.(Ser592Phefs*9) (Het);  c.6348dupT, p.(Lys2117*) (Het) | 2 months | IQ 99 (8 years 9 months) | ^7^ |
| Patient 9 | c.2935delA, p.(Arg979Glyfs*45) (Hom) | 4 months | Developmental delay (4 years) | ^7^ |
| Patient 10 | c.470C>G, p.(Ser157*) (Het);  g.6999443_6999910del,  p.(Phe1462Lysfs*1) (Het) | 3-4 months | Developmental delay (5 years 6 months) | ^7^ |
| Patient 11 | c.2935delA, p.(Arg979Glyfs*45) (Hom) | 3-4 months | Developmental delay (3 years 6 months) | ^7^ |
| Patient 12 | c.4663+1G>C (Het);  c.1404_1405delA, p.(Gly469Alafs*5) (Het) | 3-4 months | Developmental delay (12 years) | ^7^ |
| Patient 13 | c.2935delA, p.(Arg979Glyfs*45) (Het);  c.2616delG; p.(Lys872Asnfs*23) (Het) | 3-4 months | Developmental delay (2 years) | ^7^ |
| Patient 14 | c.8761C>T, p.(Arg2921*) (Het);  g.6942238_6943401del, p.(Val2929Serfs?) (Het) | 2-3 months | Developmental delay (1 years 6 months) | ^7,8^ |
| Patient 15 | c.164A>T, p.(His55Leu) (Het);  c.2108C>T, p.(Ala703Val) (Het) | 2-3 months | IQ 118 (9 years 6 months) | ^7^ |
| Patient 16 | c.3919C>T; p.(Arg1307*) (Hom) | 4-5 months | Developmental delay (7 years 6 months)  Attended higher education. Running own business (22 years) | ^7^ |
| Patient 17 | c.2935delA, p.(Arg979Glufs*45) (Hom) | 4-5 months | Developmental delay (16 years) | ^7^ |
| DDD Study | c.362_363del, p.(Tyr121Cysfs*4) (Hom) | 10 years | Normal intelligence (10 years) | ^9^ |
| DDD Study | c,5213del, p.(Lys1738Ser*9) (Het);  c.891C>A, p.(Cys297*) (Het) | N/A | N/A | ^9^ |
| Patient 1 | c.2344C>T, p. (Arg782*) (Het); c.5512C>T, p.(Gln1838*) (Het) | 3 years | N/A | ^10^ |
| Patient 1 | c.7160G>T; p.(Trp2387*) (Hom) | 2 years 6 months | Developmental delay (2 years 6 months) | ^11^ |
| Patient 1 | c.4171_4172delAG, p.(Arg1391fs*19) (Hom) | 2 years (OMA), 18 years brain MRI | Fluent in 3 languages, normal intelligence, studying within higher education college (18 years) | (Personal communication) |

,

**Supplementary Figure 1. Normal brain MRI imaging**


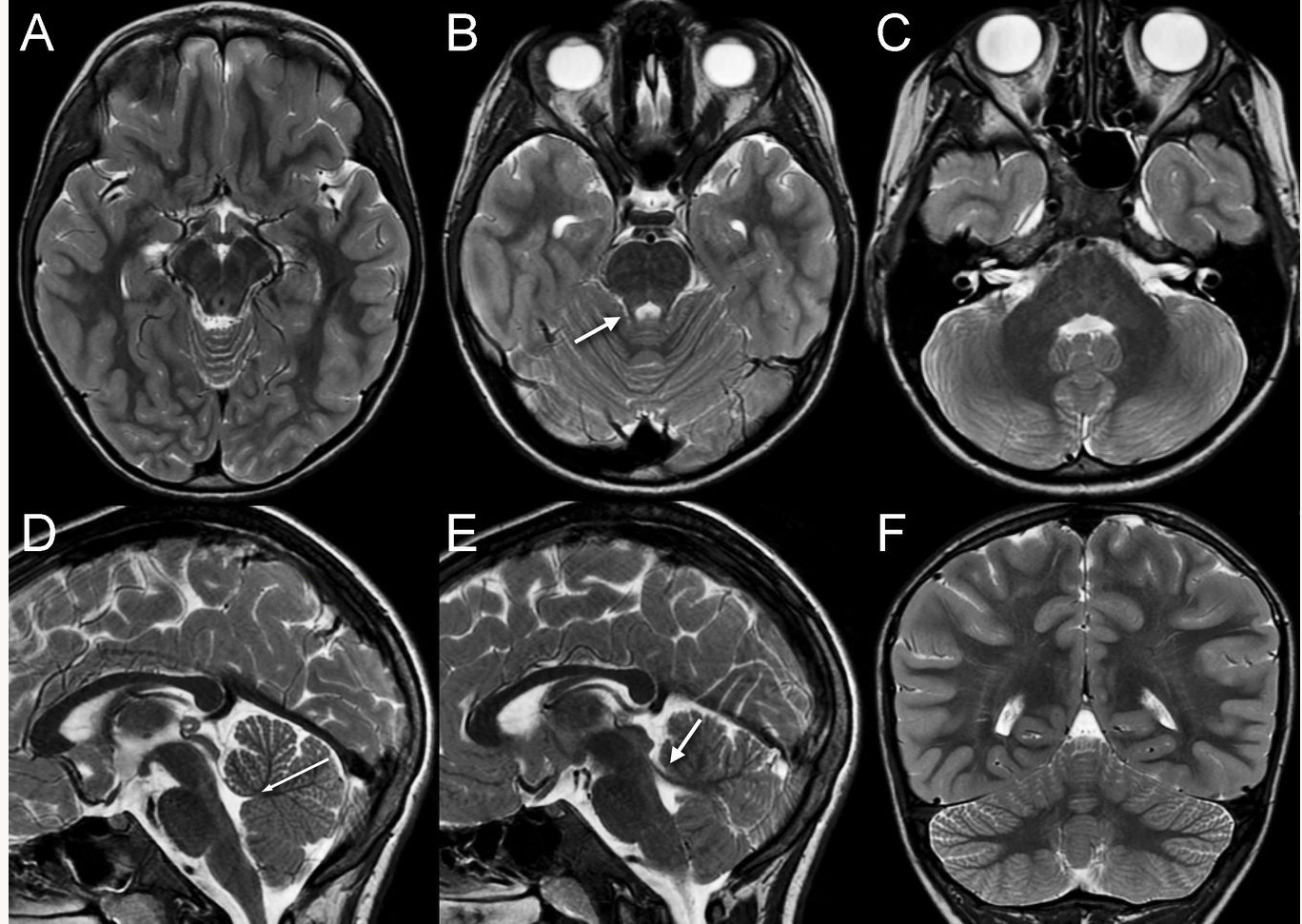


All images are T2w MRI. (A) Axial view at level of upper vermis showing regular folia. (B) Axial view showing normal dimensions of superior cerebellar peduncles (arrow). (C) Axial view at the level of normal shaped fourth ventricle. (D) Mid-sagittal view showing normal tent-like shape of fourth ventricle and a normal position of the fastigium (arrow). (E) Para-sagittal view showing normal sized and angulated superior cerebellar peduncle (arrow). (F) Coronal views showing normal white matter arborisation and regular vermis.

**Supplementary Figure 2. Typical brain MRI imaging in Poretti-Boltshauser syndrome (*LAMA1* mutations)**

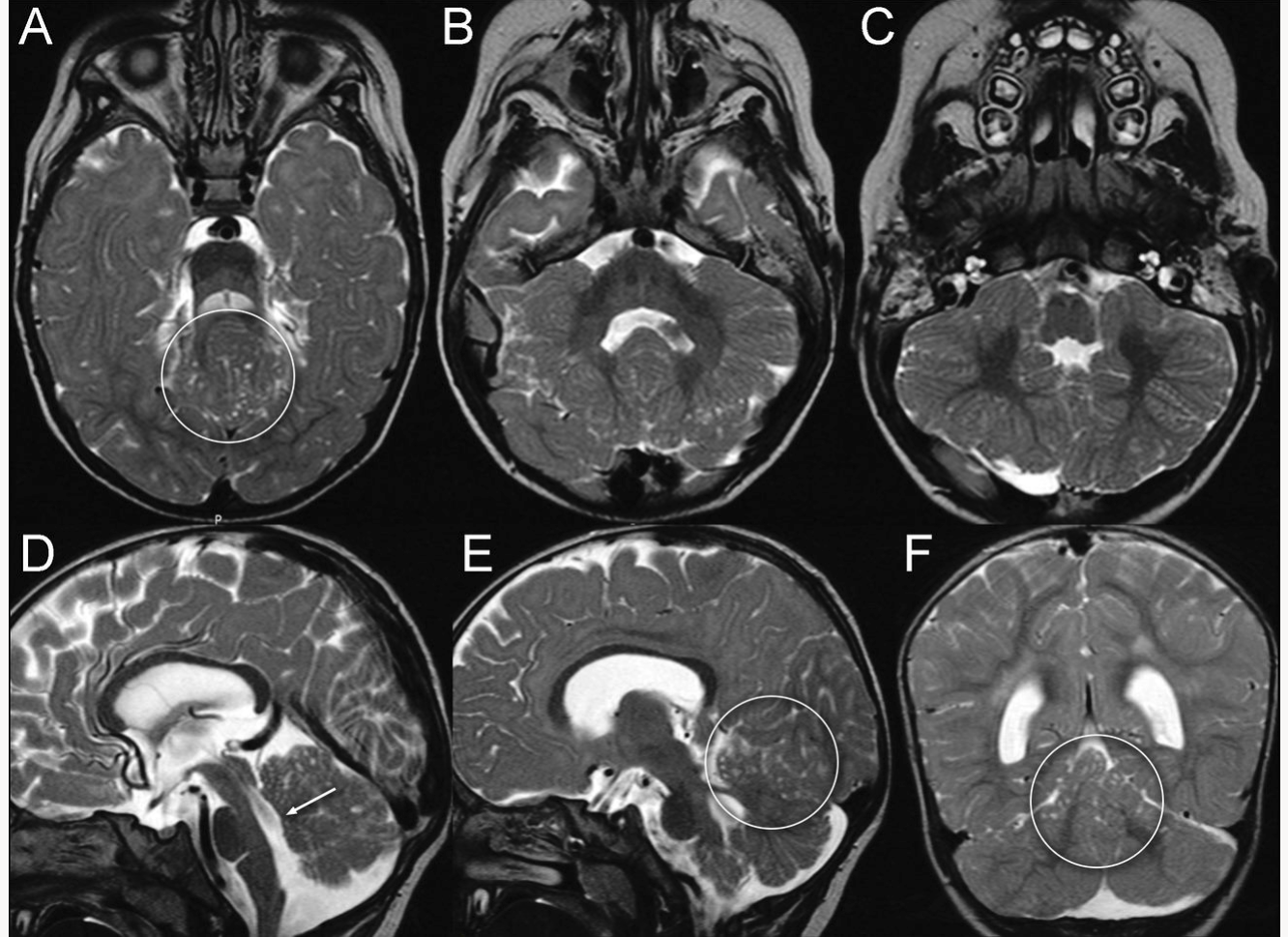


All images are T2w MRI. (A) Axial view at the level of the upper vermis showing no foliation but multiple small cysts (within circle). (B) Axial view demonstrating abnormal shape of fourth ventricle, abnormal white matter arborisation, and multiple small cysts. (C) Axial view at the level of the medulla oblongata showing marked dysplasia, i.e., irregular folial and white matter anatomy. (D) Mid-sagittal view demonstrating rhomboid-shaped fourth ventricle (arrow), absent vermis foliation, and multiple small cysts in the upper vermis. (E) Para-sagittal view: multiple small cysts in upper vermis (within circle). (F) Coronal views showing multiple cysts (within circle) and irregular architecture (compare with Figure S1 (F)).

**Supplementary Figure 3. Typical brain MRI imaging in Joubert syndrome (*CEP290* mutations)**


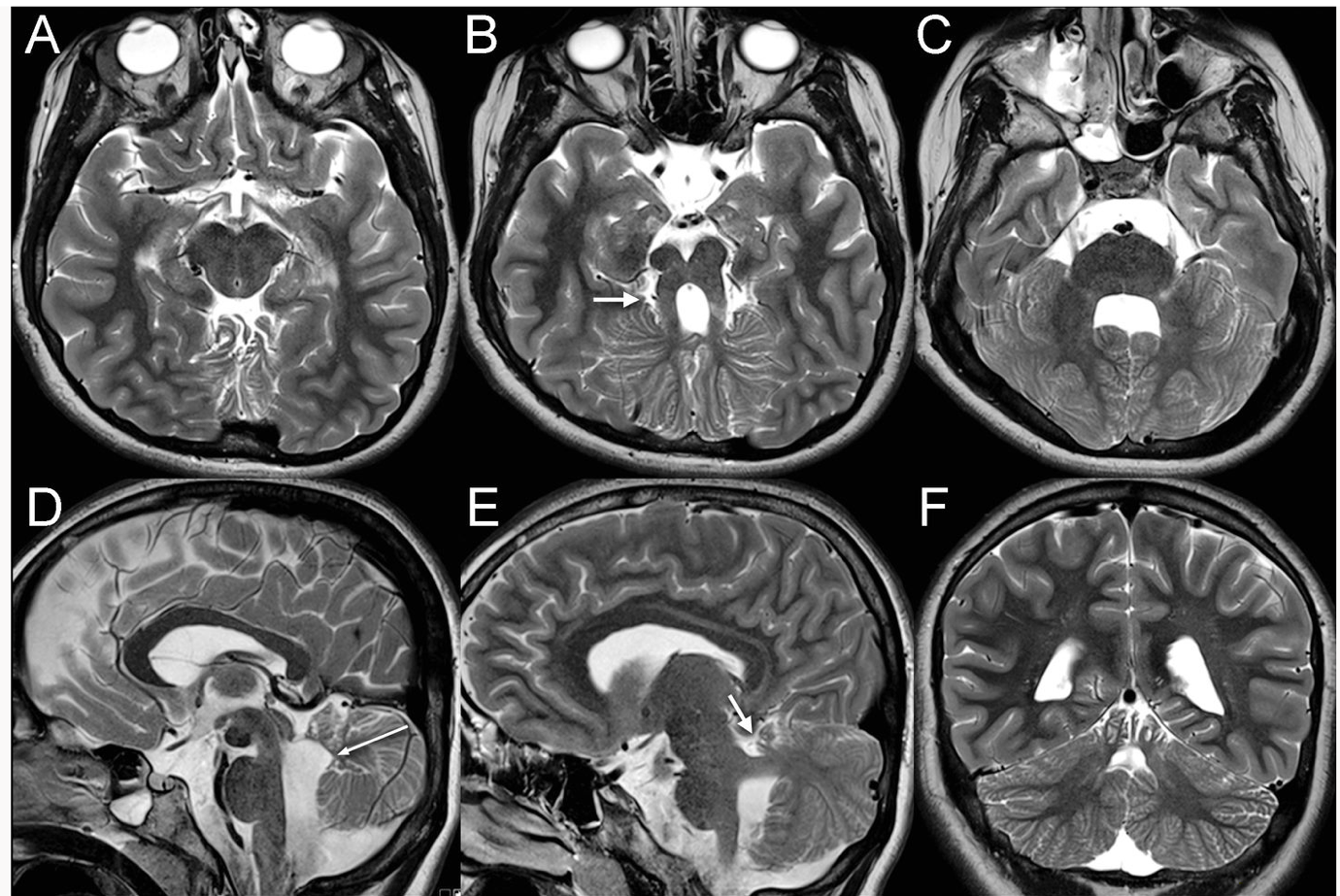


All images are T2w MRI. (A) Axial view at the level of the upper vermis showing irregular arrangement of folia. (B) Axial view at the level of the markedly thickened and elongated superior cerebellar peduncles (arrow) resulting in a “Molar Tooth Sign”. The crown of the tooth is asymmetric, a common finding. (C) Axial view at the level of the fourth ventricle showing a “bat-wing” shape. Posteriorly there is a small cleft between the cerebellar hemispheres due to vermis hypoplasia. (D) Mid-sagittal view showing abnormal shape of the fourth ventricle (compare with normal MRI (Figure S1) and cranial dislocation of fastigium (arrow). (E) Para-sagittal views demonstrating markedly thickened superior cerebellar peduncle with a horizontal course (arrow). (F) Coronal view showing irregular upper vermis with clefts.

**Supplementary Figure 4. Typical brain MRI imaging in Joubert syndrome (*INPP5E* mutations)**


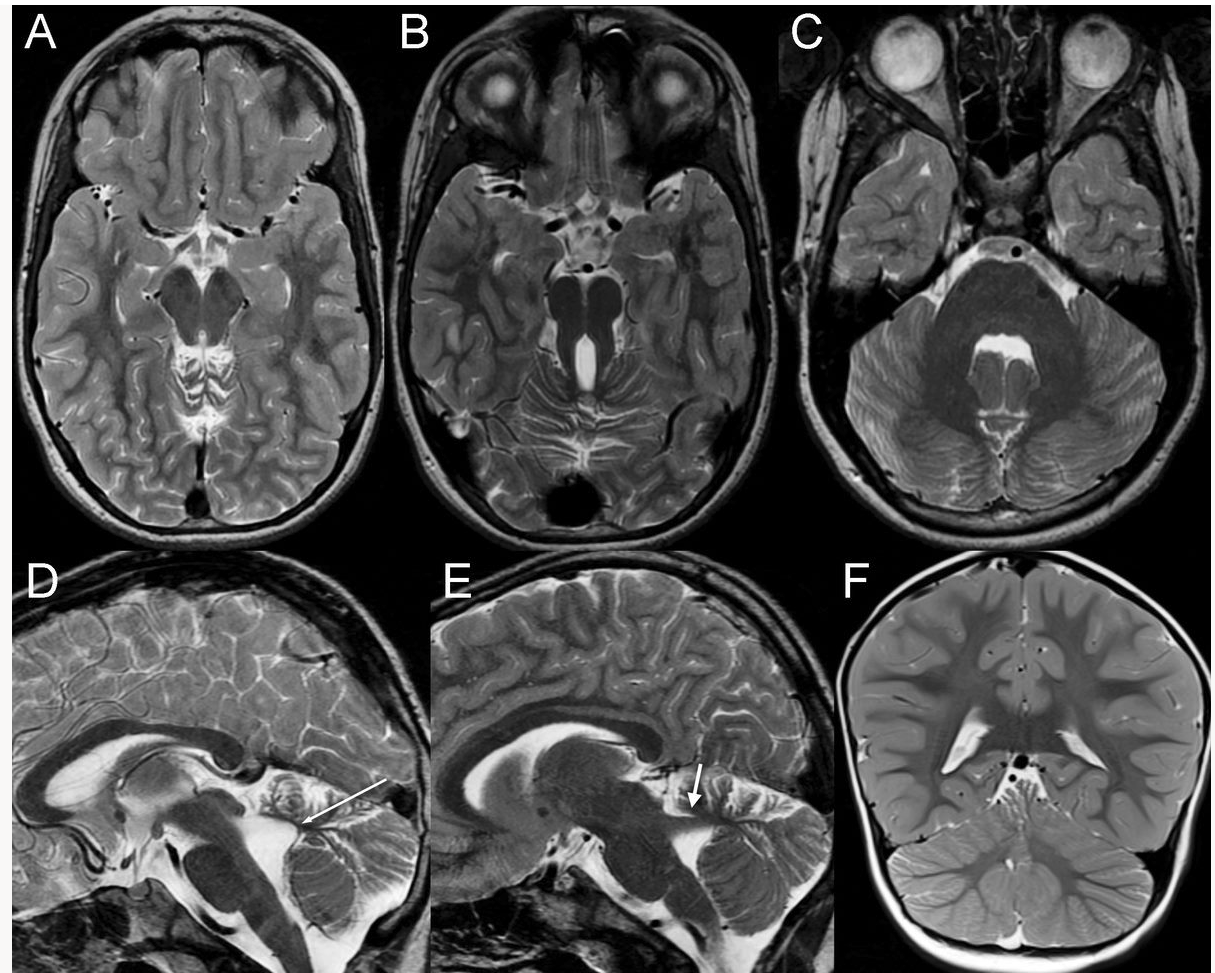


All images are T2w MRI. (A) Axial view at the level of the upper vermis showing irregular arrangement of folia. (B) Axial view at the level of the markedly thickened and elongated superior cerebellar peduncles (arrow) resulting in a “Molar Tooth Sign”. (C) Axial view at the level of the fourth ventricle showing a “bat-wing” shape. (D) Mid-sagittal view showing abnormal shape of the fourth ventricle (compare with normal MRI (Figure S1) and cranial dislocation of fastigium (arrow). (E) Para-sagittal views demonstrating markedly thickened superior cerebellar peduncle with a horizontal course (arrow). (F) Coronal view showing irregular upper vermis with clefts.


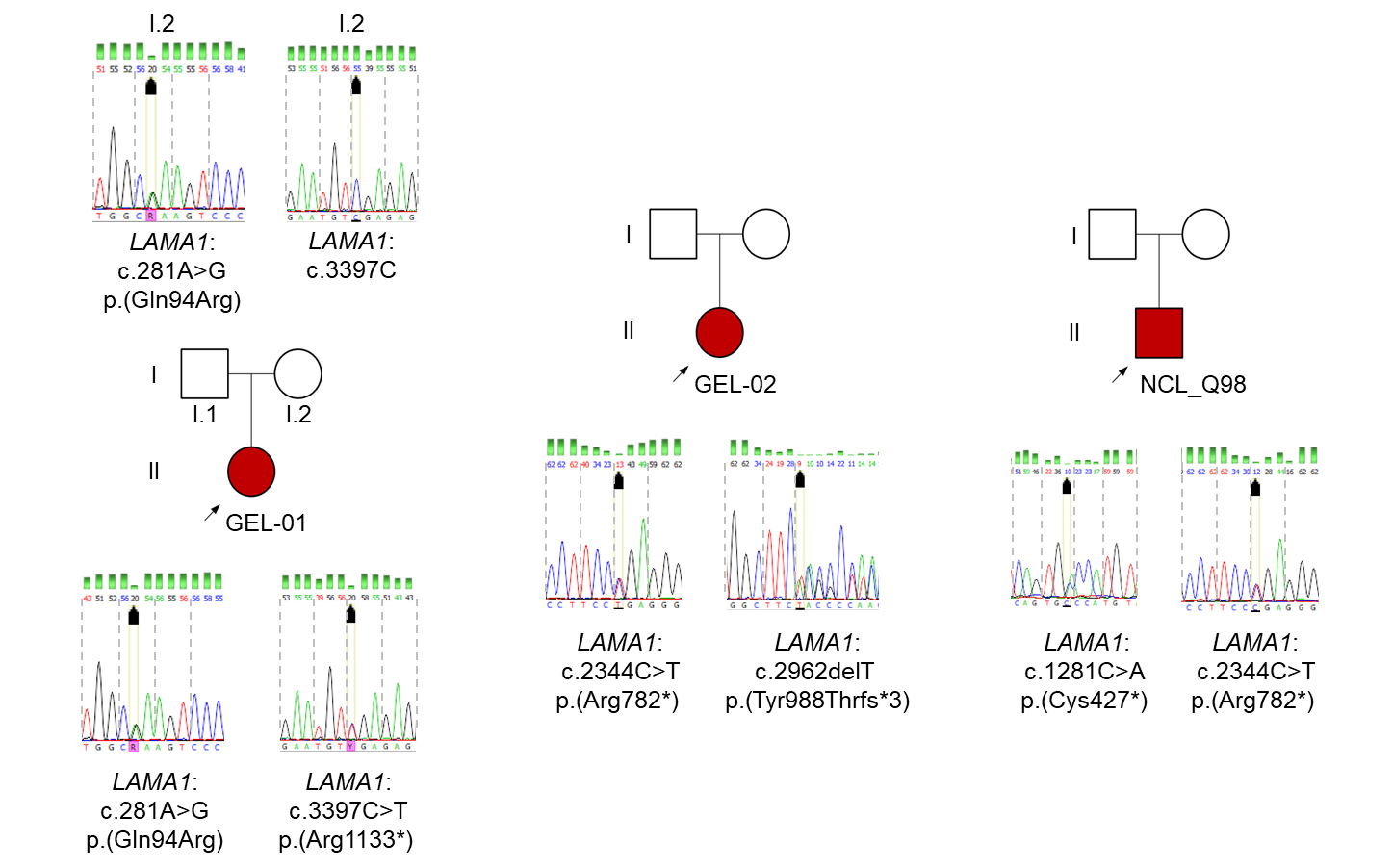


**Supplementary Figure 5. Sanger chromatogram confirmation of pathogenic *LAMA1* variants**

**Supplementary Figure 6. In silico modelling of the *LAMA1* missense allele Gln94Arg**


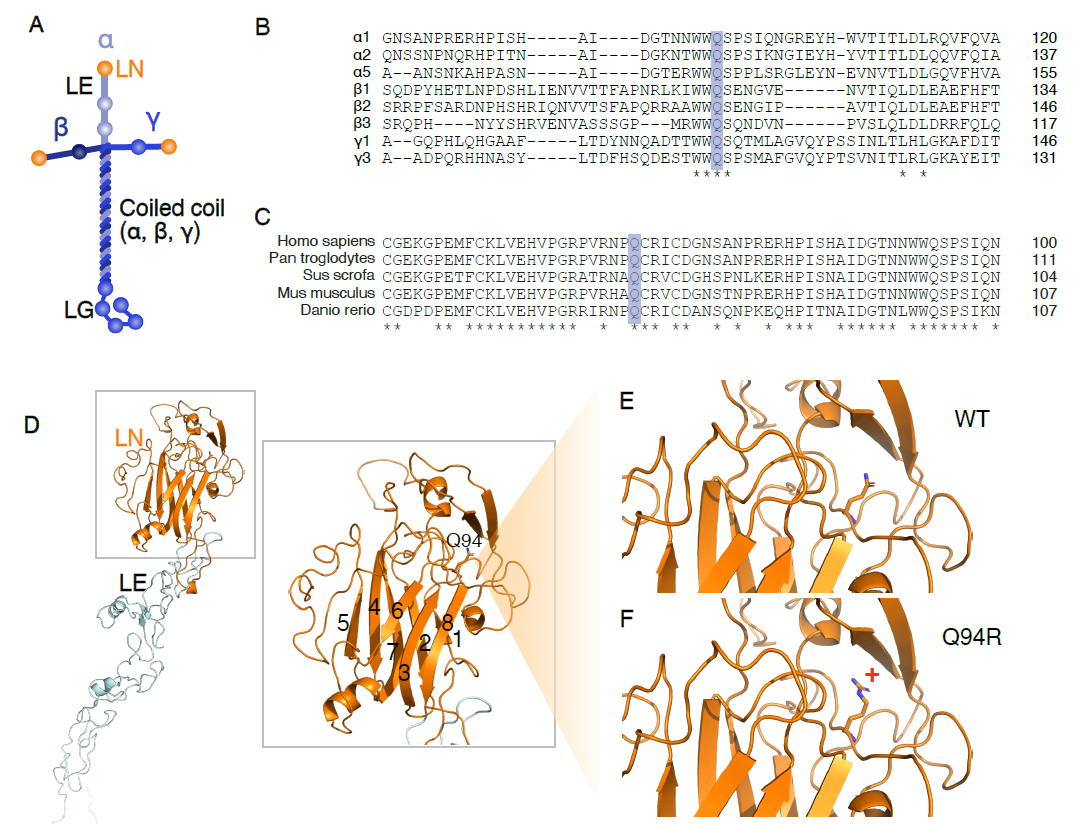


The laminin α1 Q94 residue is highly conserved and falls in the LN domain. (A) Schematic diagram of the asymmetric cross-shaped laminin molecule, consisting of three short arms α (pale blue), β (dark blue), and γ (mid-blue) and a single long arm: a coiled coil of α,β,γ. LN, N-terminal domain (orange); LE, epithelial growth factor (EGF)-like repeats interrupted by globular domains (blue); LG, C-terminal globular domains. (B) Multiple sequence alignment of human laminin isoforms containing a single LN domain. The Q94 residue is highlighted in blue. *, Conserved residue. C, Multiple sequence alignment of laminin α1 in vertebrates. The Q94 residue is highlighted in blue. *, Conserved residue. (D) Homology model of human laminin α1 based upon structural homology to mouse laminin β1 (PDB 4AQS). A zoomed in panel of the LN domain (orange) shows the 8 βstrands of the “jelly roll” fold (numbered) and the position of the Q94 residue. (E) Zoomed in image of the Q94 residue in the wild-type (WT) structure. (F) *In silico* mutagenesis of laminin α1 Q94R, resulting in the presence of a positively charged arginine residue in the LN domain.

**Supplementary Figure 7. Brain MRI of case GEL-01**


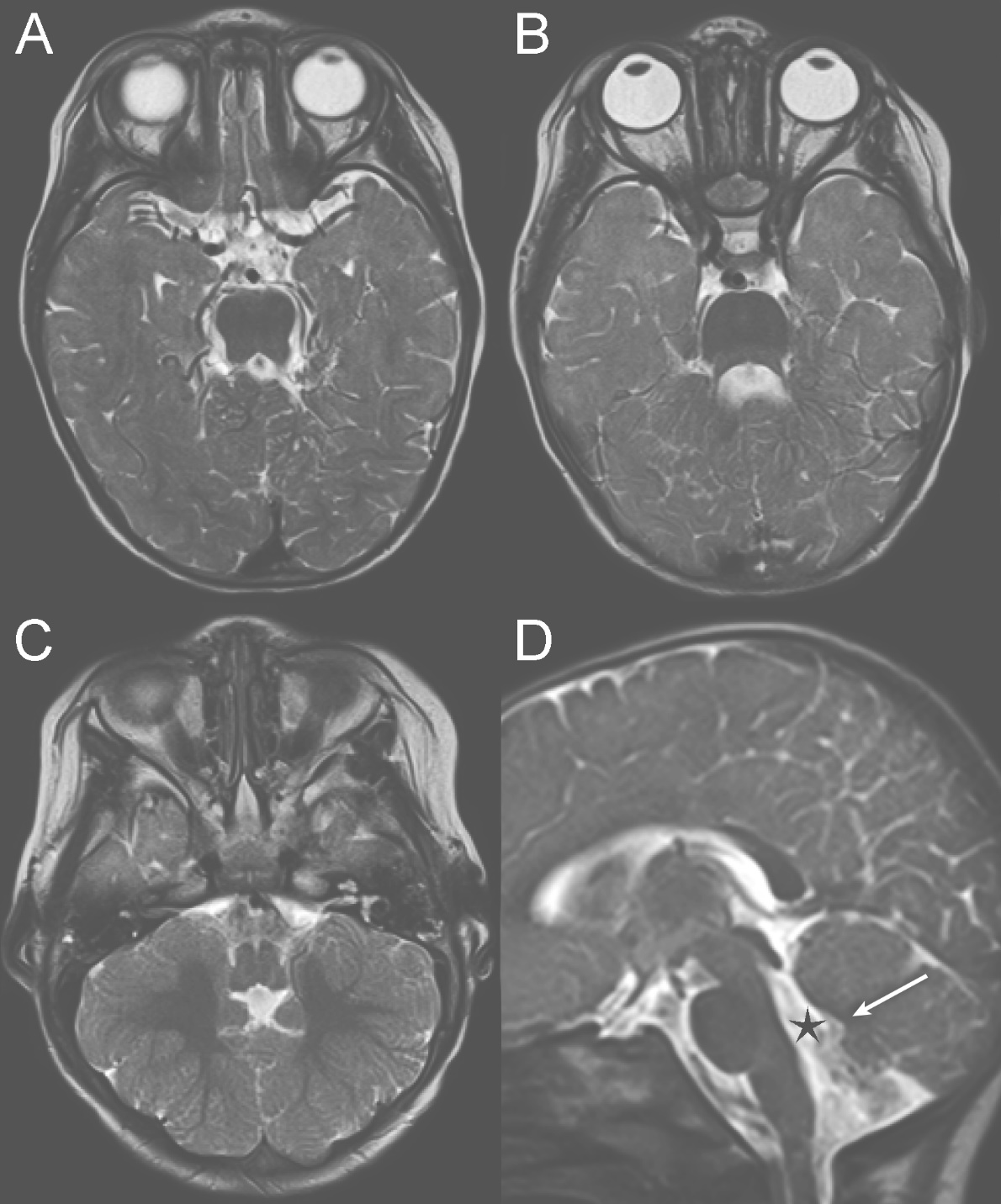


(A) Axial view at the level of the upper vermis showing dysplastic vermis and a few small cysts. (B) Axial view at the level of the enlarged and abnormally shaped fourth ventricle and splayed superior cerebellar peduncles. (C) Axial cut at the level of the medulla oblongata demonstrating dysplasia, i.e., abnormal white matter and folial anatomy. (D) Sagittal section demonstrating an abnormally shaped fourth ventricle (asterisk), the fastigium is caudally dislocated (arrow).

**Supplementary Figure 8. Brain MRI of case GEL-02**


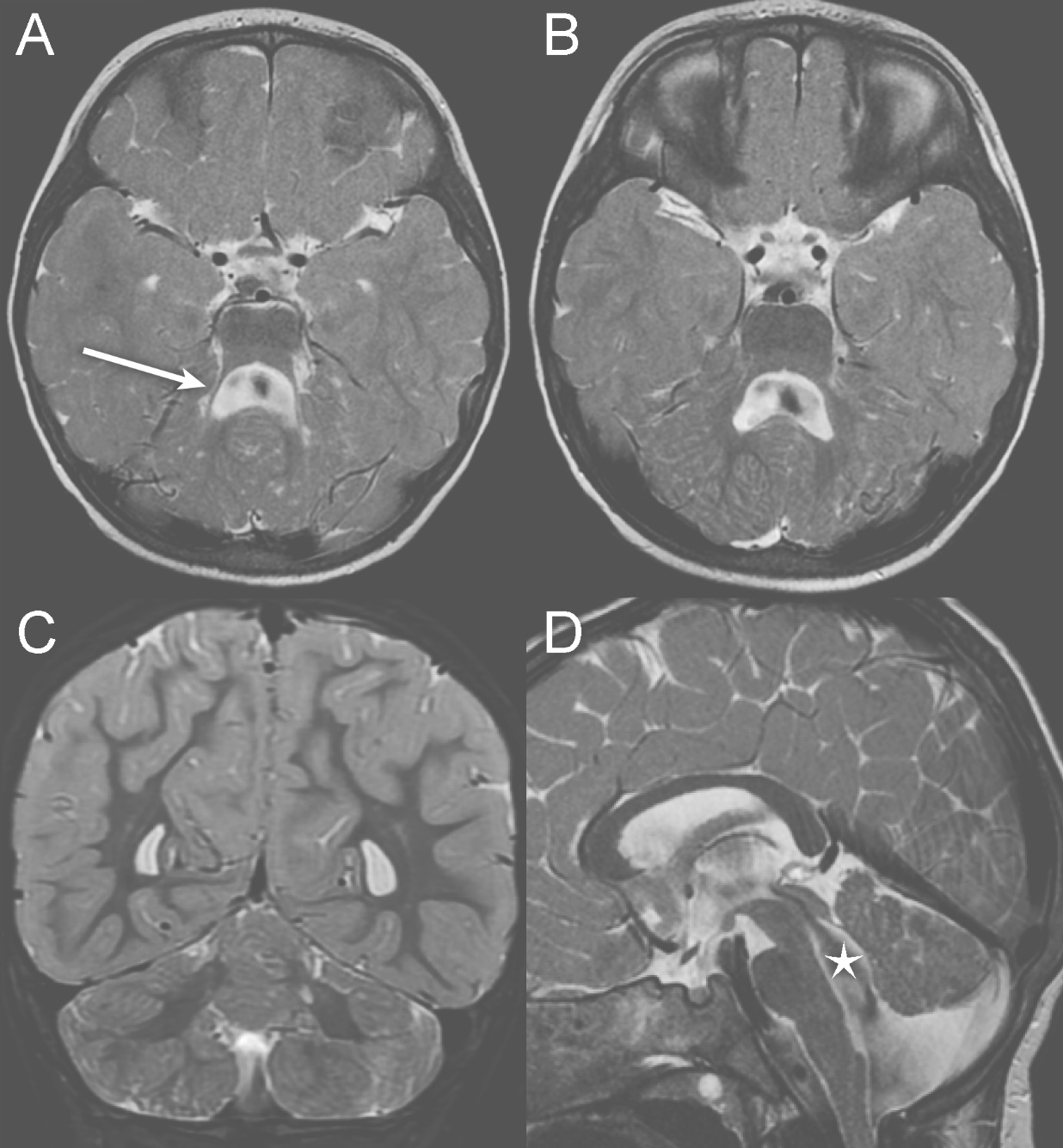


(A) Axial view at the level of the upper vermis showing thin splayed superior cerebellar peduncles and multiple small cysts behind. (B) Axial view demonstrating an abnormal shape and size of the fourth ventricle. (C) Coronal view showing disorganized white matter arborisation and foliation. (D) Sagittal view showing a rhomboid shaped fourth ventricle (asterisk), the vermis is not lobulated. (Flowing cerebro-spinal fluid leads to black signal in the aqueduct and fourth ventricle)

**Supplementary Figure 9. Brain MRI of case NCL_Q98**


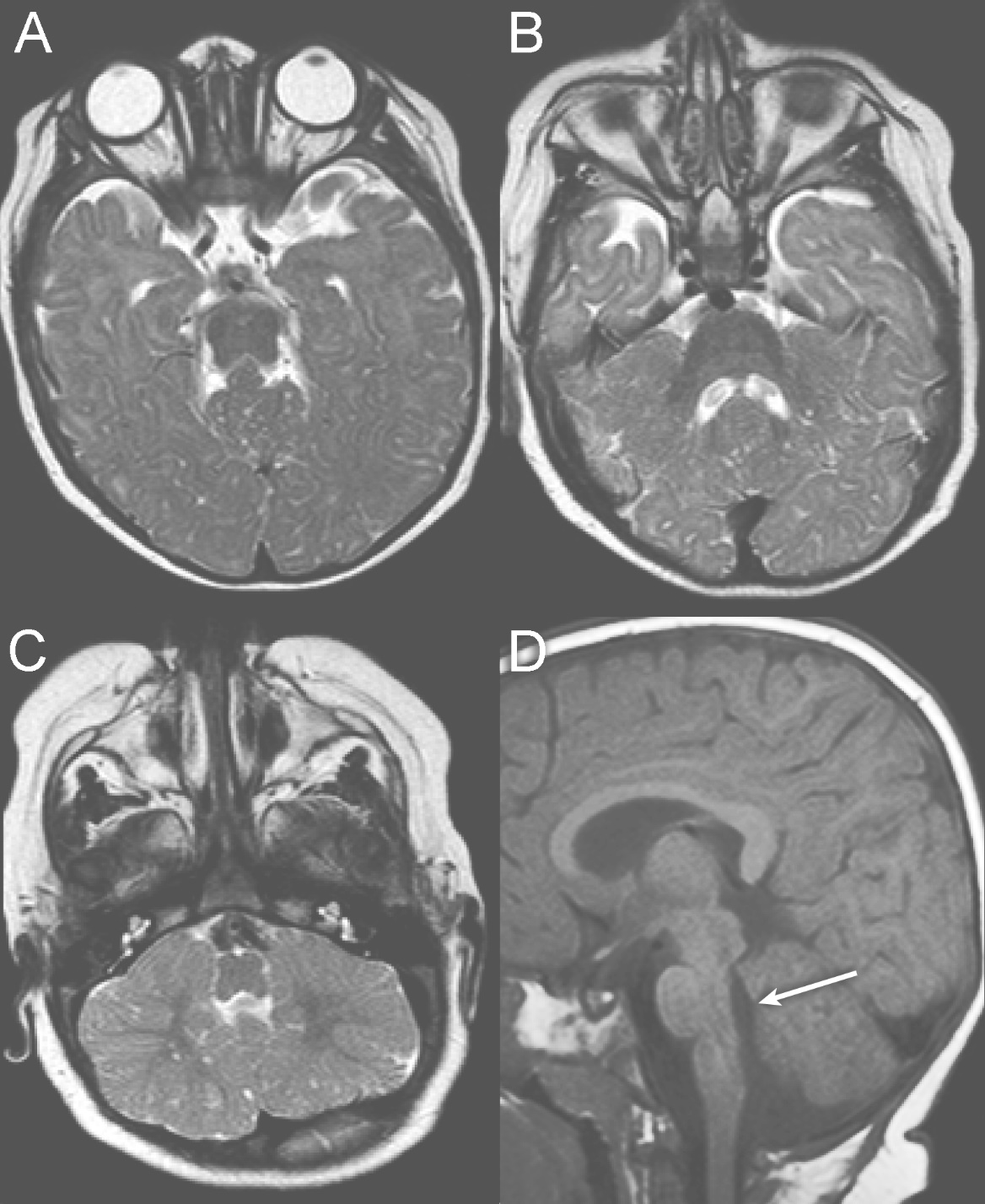


(A) Axial view at the level of the upper vermis (projected behind the brainstem) with multiple small cysts. (The cerebral white matter is not yet myelinated, appropriate for young age). (B) Axial cut at the level of the abnormal wide fourth ventricle, with multiple cysts in the vermis. (C) Axial section at the level of the medulla oblongata demonstrating dysplasia and small cysts. (D) Sagittal (T1) view showing a small flat ventricle (arrow).

**Supplementary References**

1. Epting D, Senaratne LDS, Ott E *et al*: Loss of CBY1 results in a ciliopathy characterized by features of Joubert syndrome. *Human mutation* 2020; **41:** 2179-2194.

2. Aldinger KA, Mosca SJ, Tétreault M *et al*: Mutations in LAMA1 cause cerebellar dysplasia and cysts with and without retinal dystrophy. *American journal of human genetics* 2014; **95:** 227-234.

3. Vilboux T, Malicdan MC, Chang YM *et al*: Cystic cerebellar dysplasia and biallelic LAMA1 mutations: a lamininopathy associated with tics, obsessive compulsive traits and myopia due to cell adhesion and migration defects. *Journal of medical genetics* 2016; **53:** 318-329.

4. Marlow E, Chan RVP, Oltra E, Rusu I, Gupta MP: Retinal Avascularity and Neovascularization Associated With LAMA1 (laminin1) Mutation in Poretti-Boltshauser Syndrome. *JAMA ophthalmology* 2018; **136:** 96-97.

5. Banerjee A, Vyas S, Sankhyan N: Cerebellar Cysts and Dysplasias: More Diagnoses to Consider. *Pediatric neurology* 2019; **98:** 91-92.

6. Elmas M, Gogus B, Solak M: Understanding What You Have Found: A Family With a Mutation in the LAMA1 Gene With Literature Review. *Clinical medicine insights Case reports* 2020; **13:** 1179547620948666.

7. Micalizzi A, Poretti A, Romani M *et al*: Clinical, neuroradiological and molecular characterization of cerebellar dysplasia with cysts (Poretti-Boltshauser syndrome). *European journal of human genetics : EJHG* 2016; **24:** 1262-1267.

8. Masson R, Piretti E, Pellegrin S *et al*: Early-onset head titubation in a child with Poretti-Boltshauser syndrome. *Neurology* 2017; **88:** 1478-1479.

9. Firth HV, Richards SM, Bevan AP *et al*: DECIPHER: Database of Chromosomal Imbalance and Phenotype in Humans Using Ensembl Resources. *American journal of human genetics* 2009; **84:** 524-533.

10. Cai CX, Go M, Kelly MP, Holgado S, Toth CA: OCULAR MANIFESTATIONS OF PORETTI-BOLTSHAUSER SYNDROME: FINDINGS FROM MULTIMODAL IMAGING AND ELECTROPHYSIOLOGY. *Retinal cases & brief reports* 2020.

11. Alahmadi AS, Badawi AH, Magliyah MS, Albakri A, Schatz P: Poretti-Boltshauser syndrome: a rare differential diagnosis to consider in pediatric high myopia with retinal degeneration. *Ophthalmic genetics* 2021; **42:** 96-98.
