# Supplementary material for: Identification of LAMA1 mutations ends diagnostic odyssey and has prognostic implications for patients with presumed Joubert syndrome
Source: Brain Commun. 2021 Jul 16;3(3):fcab163. doi: 10.1093/braincomms/fcab163 (PMC8374969; doi:10.1093/braincomms/fcab163)
Supplement: fcab163_Supplementary_Data [file fcab163_Supplementary_Data.zip › Manuscript_original_submission.pdf]

OXFORD UNIVERSITY PRESS | Brain Communications

Identification of LAMA1 mutations ends diagnostic odyssey and has prognostic implications for patients with presumed Joubert syndrome

Journal:	*Brain Communications*
Manuscript ID	BRAINCOM-2021-135
Manuscript Type:	Original Article
Date Submitted by the Author:	19-Apr-2021
Complete List of Authors:	Powell, Laura; Newcastle University, Translational and Clinical Research Institute<br>Olinger, Eric; Newcastle University, Translational and Clinical Research Institute<br>Wedderburn, Sarah; NHS Greater Glasgow and Clyde, Clinical Genetics<br>Ramakumaran, Vijayalakshmi; Oxford University Hospitals NHS Foundation Trust, Clinical Genetics<br>Kini, Usha; Department of Clinical Genetics, Oxford Radcliffe NHS Trust,<br>Clayton-Smith, Jill; The University of Manchester, Manchester Centre for Genomic Medicine, University of Manchester, St Mary's Hospital, Manchester, United Kingdom<br>Ramsden, Simon; Central Manchester University Hospitals NHS Foundation Trust, Manchester Centre for Genomic Medicine<br>Rice, Sarah; Newcastle University, Biosciences Institute<br>Barroso-Gil, Miguel; Newcastle University, Translational and Clinical Research Institute<br>Wilson, Ian; Newcastle university,<br>Cowley, Lorraine; Newcastle Upon Tyne Hospitals NHS Foundation Trust, Northern Genetics Service<br>Johnson, Sally; Newcastle Upon Tyne Hospitals NHS Foundation Trust, Great North Children's Hospital<br>Harris, Elizabeth; Newcastle Upon Tyne Hospitals NHS Foundation Trust, Northern Genetics Service<br>Montgomery, Tara; Newcastle Upon Tyne Hospitals NHS Foundation Trust, Northern Genetics Service<br>Bertoli, Marta; Newcastle Upon Tyne Hospitals NHS Foundation Trust, Clinical Genetics<br>Boltshauser, Eugen; University Children's Hospital , Department of Pediatric Neurology<br>Sayer, John; Newcastle University, Translational and Clinical Research Institute; Newcastle Upon Tyne Hospitals NHS Foundation Trust, Renal Services
Keywords:	joubert, LAMA1, Ataxia, Molecular genetics

Identification of *LAMA1* mutations ends diagnostic odyssey and has prognostic implications for patients with presumed Joubert syndrome

Laura Powell[1], Eric Olinger[1], Sarah Wedderburn[2], Vijayalakshmi Salem Ramakumaran[3], Usha Kini[3], Jill Clayton-Smith[4,5], Simon C. Ramsden[4], Sarah J. Rice[6], Miguel Barroso-Gil[1], Ian Wilson[6], Lorraine Cowley[7], Sally Johnson[1,8], Elizabeth Harris[7], Tara Montgomery[7], Marta Bertoli[7], Genomics England Research Consortium, Eugen Boltshauser[9] and John A. Sayer[1,10,11]

[1]Translational and Clinical Research Institute, Faculty of Medical Sciences, Newcastle University, Central Parkway, Newcastle upon Tyne, NE1 3BZ, United Kingdom
[2]Clinical Genetics, NHS Greater Glasgow and Clyde, United Kingdom
[3]Clinical Genetics, Oxford University Hospital, Oxford, United Kingdom
[4]Manchester Centre for Genomic Medicine, Manchester University Hospitals NHS Foundation Trust, Oxford Road, Manchester M13 9WL, United Kingdom
[5]Division of Evolution and Genomic Sciences School of Biological Sciences University of Manchester, M13 9PL
[6]Bioscience Institute, Faculty of Medical Sciences, Newcastle University, Central Parkway, Newcastle upon Tyne, NE1 3BZ, United Kingdom
[7]Clinical Genetics, Northern Genetics Service, Newcastle upon Tyne Hospitals NHS Foundation Trust, Central Parkway, Newcastle upon Tyne, NE1 3BZ, United Kingdom
[8]Paediatric Nephrology, The Great North Children's Hospital, Royal Victoria Infirmary, Queen Victoria Road, Newcastle upon Tyne, NE1 4LP, United Kingdom
[9]Paediatric Neurology (Emeritus), Children's University Hospital, Zürich, Switzerland
[10]Renal Services, Newcastle Upon Tyne Hospitals NHS Foundation Trust, Newcastle upon Tyne, NE7 7DN, United Kingdom
[11]NIHR Newcastle Biomedical Research Centre, Newcastle upon Tyne, NE4 5PL, United Kingdom

Corresponding author:
Prof John A. Sayer, Professor of Renal Medicine
Translational and Clinical Research Institute, Faculty of Medical Sciences, Newcastle University, Central Parkway, Newcastle upon Tyne, NE1 3BZ, United Kingdom
Email: john.sayer@newcastle.ac.uk

For Review Only

Abstract

Paediatric neurology syndromes are a broad and complex group of conditions with a large spectrum of clinical phenotypes. Joubert syndrome is a genetically heterogeneous neurological ciliopathy syndrome with molar tooth sign as the neuroimaging hallmark. We reviewed the clinical, radiological and genetic data for several families with a clinical diagnosis of Joubert syndrome but negative genetic analysis. We detected biallelic pathogenic variants in *LAMA1*, including novel alleles, in each of the four cases we report, thereby establishing a firm diagnosis of Poretti-Boltshauser syndrome. Analysis of brain MRI revealed cerebellar dysplasia and cerebellar cysts, associated with Poretti-Boltshauser syndrome and the absence of typical molar tooth signs. Using large UK patient cohorts the relative prevalence of Joubert sydrome as a cause of intellectual disability was 0.2% and Poretti-Boltshauser was 0.02%. We conclude that children with congenital brain disorders that mimic Joubert syndrome may have a delayed diagnosis due to poor recognition of key features on brain imaging and the lack of inclusion of *LAMA1* on molecular genetic gene panels. We advocate the inclusion of *LAMA1* genetic analysis on all intellectual disability and Joubert syndrome gene panels and promote a wider awareness of the clinical and radiological features of these syndromes.

For Review Only

Key words: Joubert syndrome; Poretti-Boltshauser syndrome; *LAMA1*; cerebellar cysts; cerebellar dysplasia; molecular genetics; molar tooth sign.

.

Introduction

Poretti-Boltshauser syndrome (PTBHS) (OMIM #615960) is a rare neuro-ophthalmological disease with phenotypes that may include a non-progressive cerebellar ataxia, delayed motor and language development and intellectual disability. There are additional ophthalmological phenotypes associated with this condition that may include ocular motor apraxia, myopia, strabismus and retinal dystrophy. On brain imaging cerebellar dysplasia, cerebellar cysts and cerebellar vermis hypoplasia are typically seen. The syndrome was first described in 2014 where the features of 7 children (from 5 unrelated families) were described [1]. Subsequently, the underlying molecular defect was shown to be biallelic truncating or splice site mutations in *LAMA1* [2] which encodes the Laminin alpha-1 protein. Since these initial reports of PTBHS phenotypes there has been only a handful of publications detailing additional cases [3-7]. This suggests either that the disease remains ultra-rare, or that cases of PTBHS are not being recognised and are being incorrectly labelled. Another rare condition affecting the cerebellum is Joubert syndrome, a syndrome characterised clinically by a wide array of features including hypotonia, abnormal breathing patterns, ocular motor apraxia, ataxia and itellectual disability. Additional features include retinal dystrophy, nephronophthisis, liver fibrosis and skeletal dysplasia [8]. The condition was described in 1969 by Marie Joubert [9] and subsequently several more families with episodic hyperpnea, abnormal eye movements and ataxia were noted [10]. Joubert syndrome has an estimated prevalence of 1/55,000–1/200,000 [11-13]. Brain MRI in Joubert syndrome typically reveals a molar tooth sign secondary to a deep interpeduncular fossa and elongation of the superior cerebellar peduncles [10]. Joubert syndrome is clinically heterogeneous [13-16] and there are currently 38 known genetic causes of Joubert syndrome [8].

Next generation sequencing (NGS) approaches have over the past decade rapidly improved our ability to diagnose rare inherited diseases [16-20] and these have been applied to patients with suspected Joubert syndrome.

Here we review the clinical and imaging details of a child with developmental delay and ataxia, who had been labelled as having Joubert syndrome. We were able to establish a

For Review Only

diagnosis of PTBHS following review of imaging and WES approaches. This prompted us to identify another three cases where similar diagnostic pitfalls led to diagnostic delays.

For Review Only

Methods

Ethical Approvals and Patients Inclusion and Clinical Evaluation

This study was approved by the North East -Newcastle & North Tyneside 1 Research Ethics Committee (18/NE/350) and the Genomics England 100,000 Genomes Project was approved by the Health Research Authority Research Ethics Committee East of England – Cambridge South (REC Ref 14/EE/1112). All patients had an initial evaluation where clinical features were suggestive of Joubert syndrome with neurological phenotypes. Written and informed consent was obtained from patients and family members involved in this study.

Genomics England 100,000 Genomes Project

Whole genome sequencing (WGS) was performed through the Genomics England 100,000 Genomes Project (GE100KGP). All participants in the 100,000 Genomes Project have provided written consent to provide access to their anonymised clinical and genomic data for research purposes.

Deciphering Developmental Disorders (DDD) Study

13,451 individuals with severe, undiagnosed developmental disorders were recruited from 24 regional genetics services within the United Kingdom National Health Service and the Republic of Ireland. Families gave informed consent to participate, and the study was approved by the UK Research Ethics Committee (10/H0305/83 granted by the Cambridge South Research Ethics Committee, and GEN/284/12 granted by the Republic of Ireland Research Ethics Committee). Details on sample collection and genetic analysis pipelines have been described previously [21]. Genetic variants and linked phenotypic data have been accessed via DECIPHER (https://www.deciphergenomics.org/) (last access 30/03/21).

Variants Validation by Sanger Sequencing

For Review Only

Sanger sequencing was utilized to confirm variants and their segregation from both parents where DNA samples where available. PCR amplification was performed using *Taq* PCR master mix (Qiagen) kit, as per the manufacturer instructions.

For Review Only

Results

Patients Characteristics

The index family with proband (NCL_Q73) was a child was 3 years of age and had been labelled as Joubert syndrome given her clinical features of ataxia, ocular motor apraxia and a possible molar tooth sign detected on brain MRI imaging (Table 1, Figure 1). A previous genetic analysis using a next generation sequencing (NGS) 29 genes panel of Joubert syndrome genes was negative. Her history was reviewed as well as her brain imaging (Table 1) and features seemed to diverge from a typical Joubert syndrome presentation. In particular, the brain MRI scans showed evidence of cerebellar cysts (Figure 1, Tables S1, S2, Figures S1-S4).

Exome Sequencing Data

Following informed consent, WES was carried out for the proband and her mother in order to pursue a precise molecular genetic diagnosis. WES data was initially filtered for biallelic changes in 38 genes known to cause Joubert syndrome (Table S3) and monoallelic changes in *SUFU*. This led to no obvious underlying genetic causes in this set of genes. Filtering of variants was adjusted to examine all rare variants within the exome dataset and identified two rare and predicted pathogenic alleles in *LAMA1* (Figure 1, Table 2), which were confirmed by Sanger sequencing and segregated from each parent. Biallelic variants in *LAMA1* cause Poretti-Boltshauser syndrome (PTBHS), which would fully account for the clinical presentation and phenotype. The identification of this index case of PTBHS and the diagnostic pitfalls leading to an erroneous clinical diagnosis of Joubert syndrome led us to search for other similar mislabelled cases.

For Review Only

Identification of additional patients with undiagnosed Poretti-Boltshauser syndrome

The 100,000 Genomes Project provides a rich source of whole genome sequence (WGS) data on patients with rare disease phenotypes [22]. We searched the whole rare disease dataset (73,988 genomes) for pathogenic alleles in *LAMA1* and identified 2 probands (GEL-01 and GEL-02) with rare, pathogenic biallelic changes in *LAMA1* (Figure 1, Tables 1,2, Figure S5, S6) and brain MRI phenotypes consistent with PTBSH (Figures S7, S8). These alleles had been filtered out by the standard tiering tables and the patients' physicians had received an "exit questionnaire" suggesting no causative mutations had been identified and that the patients remained genetically unsolved. The most likely reason for this is that these patients were recruited into phenotypic groups "Congenital malformations caused by ciliopathies (Specific disease Joubert syndrome) for patient GEL-01 and "Motor disorders of the CNS" (Specific disease cerebellar hypoplasia) for patient GEL-02, neither of which contained *LAMA1* as a causative gene in the virtual gene panel applied. We have subsequently added *LAMA1* as a potential causative gene to these panels via the Genomics England PanelApp (https://panelapp.genomicsengland.co.uk) so that future cases will not be missed. Finally, we examined the DECIPHER database [23] (https://decipher.sanger.ac.uk/) for patients with *LAMA1* mutations and found an additional UK family with a proband (NCL_Q98) who had been clinically labelled Joubert syndrome at the age of 14 months based on the presence of ocular motor apraxia (OMA) and an abnormal MRI brain scan (Figure 1, Tables 1,2, Figure S9). A gene panel for Joubert syndrome had been previously performed which showed no mutations. The family was enrolled into the DDD study which had revealed a molecular genetic diagnosis of *LAMA1* mutations (Patient 271064) (Figure S5) and a clinical diagnosis was confirmed to be Poretti-Boltshauser syndrome [24].

Finally, we examined published cases of PTHBS in the literature to determine longer term outcomes for patients with this condition, given that this is a frequently asked question by both physicians and affected family members (Table S4). Data is limited but there is some

For Review Only

evidence of extremely good outcomes with adult patients with normal IQ levels, attending college and higher education and running own businesses. The caveat is that the majority of known patients with PTBHS are still children and the long-term outlook is not yet known.

Estimated prevalence of Poretti-Boltshauser syndrome and Joubert syndrome in UK patient cohorts

In order to assess the relative prevalence of molecular diagnoses of Poretti-Boltshauser syndrome and Joubert syndrome in patients with intellectual disabilities and developmental disorders, we searched the Genomics England (GEL) 100,000 Genomes Project rare disease dataset as well as Deciphering Developmental Disorders (DDD) study for cases solved with either one of the 38 known Joubert syndrome genes (Table S3) or pathogenic variants in *LAMA1*. Out of 8459 probands recruited in GEL with Human Phenotype Ontology (HPO) terms “intellectual disability” and/or “developmental delay”, 18 cases (0.2%) were molecularly solved for Joubert syndrome genes versus only 2 cases solved with *LAMA1* variants (Figure 2A). Similarly, among ~14,000 individuals with developmental disorders recruited in the UK DDD study, 26 (~0.2%) were solved for a Joubert syndrome gene and 3 cases solved with *LAMA1* variants (Figure 2B). Molecular genetics approaches, unbiased for clinical or radiological assessments, indicate thus that Poretti-Boltshauser syndrome is a considerably rarer cause of developmental disorders and intellectual disability than the collective of genes causing Joubert syndrome, at least in these UK cohorts. On the other hand, the relative contribution of *LAMA1* to developmental disorders is likely comparable to the more common among the Joubert syndrome genes, when considered individually (Figure 2).

For Review Only

Discussion

Joubert syndrome and PTBHS can present in very similar ways in *infancy,* and this may cause diagnostic uncertainty and confusion (Table S1, Figure S10). Clearly it is vital that at this time, when patients are "deviating" from normal development, appropriate investigations are planned. For multisystem syndromes such as Joubert syndrome it is desirable for an *early* diagnosis to be made, for example before the onset of end stage kidney disease. For a more limited condition there may be less urgency, but both family members and physicians will appreciate a precise diagnostic label so appropriate management plans can be put in place and the diagnostic work-up stopped. The main clinical similarities between Joubert syndrome and PTBHS are delays in motor and speech development, and ocular motor apraxia in the vast majority (Table S1). A recent observation is that autosomal dominant variant in *SUFU* cause a similar clinical phenotype in infancy [25]. We emphasise that the clinical findings in infancy do overlap but later in childhood the syndromes of Joubert syndrome and PTBHS diverge.

Regarding brain imaging, pattern recognition is required for the identification and distinction of the two syndromes. The radiologist needs high-definition images in order to make a correct diagnosis and needs to be qualified in the interpretation of neurological conditions. The field of posterior fossa anomalies may not be an area of expertise for most radiologists. This point is important as the correct interpretation of brain imaging will lead to the correct set of genetic investigations to be performed and will result in a faster, more cost-effective diagnostic odyssey for the patient.

For Joubert syndrome the MRI imaging hallmark is the molar tooth sign, but in practice there is an imaging spectrum of additional infra- and supratentorial anomalies [26]. It is noteworthy that the MTS is variable in peculiarity - from very pronounced to less marked, in the literature also called "mild MTS". Non-specialists may easily overlook mild MTS features on brain MRI. The findings of mild MTS have often been reported in Joubert syndrome associated with mutations in *NPHP1*, *C5orf42*, *FAM149B1*, *CBY1* [27]. The mild

For Review Only

MTS seen in some types of Joubert syndrome is not distinguishable from mild MTS in patients with *SUFU* variants, emphasizing the role of targeted molecular genetic investigations for these conditions. The differential diagnosis for MTS includes pontine cap dysplasia, but the clinical context and remainder of imaging are very different [28].

We would like to emphasize that there is no imaging overlap between Joubert syndrome and PTBHS (Table S2). In PTBHS brain imaging there is a “hierarchy” of findings, of which cerebellar dysplasia is the leading sign, followed by cerebellar cysts. The differential diagnosis of cerebellar dysplasia and cysts includes some severe forms of congenital muscular dystrophies however, other clinical contexts and additional MRI anomalies do not allow for diagnostic confusion.

The accessibility of WGS and WES in both research and clinical care settings has allowed huge advances in our understanding and diagnosis of rare diseases. The interpretation of rare variants identified by an NGS approach relies heavily on accurate phenotypic data provided by the referring physician. In this context, an expert clinical understanding of disease phenotypes and the corresponding disease alleles is also required to judge the pathogenicity of alleles within known or candidate genes relating to the underlying syndrome. We have demonstrated that there is a limited phenotypic overlap between Joubert syndrome and PTBHS and because of a lack of awareness of both these rare conditions it seems a pragmatic approach to add the single *LAMA1* gene to Joubert syndrome panels so this alternate diagnosis can be detected. Gene discovery in the arena of neurodevelopmental disorders is fast moving [20, 27]. Efforts to reanalyse unsolved Joubert syndrome-like patients and those with cerebellar dysplasia and cysts should be made with virtual gene panels that have been updated for all differential diagnoses. Expert multidisciplinary teams (MDT) can then be utilised to report back the alleles and their pathogenicity in context with the clinical phenotype, which can evolve as the child develops.

For Review Only

In discovering these cases of PTBHS we were struck by the profound impact the genetic diagnosis made on each of the families. In all 4 cases, there was a delay caused by incomplete genetic analysis and failure to include *LAMA1* in the gene panel or virtual gene panel applied. Due to this lack of molecular genetic diagnosis, in all 4 cases the clinical diagnosis defaulted to Joubert syndrome. The molecular genetic diagnosis of *LAMA1* mutations led to a new diagnostic label and a feeling of loss and insecurity for the families involved. A new diagnosis also has some prognostic implications. Compared to the multisystem features of Joubert syndrome, PTBHS has a much more limited phenotype. To date, no other organs aside from the brain are involved and there is no evidence for disease progression. In particular, it is not associated with the renal and liver problems which may be seen in Joubert syndrome and individuals with an established diagnosis of PTBHS do not require regular monitoring for these. Although ocular motor apraxia persists into adult life. With that in mind we tried to draw some conclusions from the older patients that have been reported in the literature. This confirmed the non-progressive nature of the PTBHS phenotype and overall favourable outcomes including some examples of normal intellectual ability and independence but we suspect this data is both incomplete and subject to bias. In line with a somewhat milder phenotype, we also detected a ~50 year old individual in gnomAD carrying a homozygous predicted loss-of-function variant in *LAMA1* (c.858+1G>T).

NGS has become more widely available for use as a diagnostic tool for the investigation of patients with cerebellar disorders. It has the potential to resolve those cases with suspected Joubert syndrome and disorders such as PTBHS which may clinically mimic Joubert syndrome in infancy. We present 4 real world examples of how a precise molecular diagnosis of *LAMA1* mutations leads to a revised diagnosis of PTBHS which allows some clarity in terms of clinical monitoring for the physician and potential long-term outcomes for the patient.

Acknowledgements

For Review Only

We thank the affected individuals, their families, and their physicians who contributed to this study. Laura Powell is funded by the Medical Research Council Discovery Medicine North Training Partnership. Eric Olinger is supported by an Early Postdoc Mobility Stipendium of the Swiss National Science Foundation (P2ZHP3_195181) and Kidney Research UK (Paed_RP_001_20180925). Miguel Barroso-Gil is funded by Kidney Research UK (ST_001_20171120) and the Northern Counties Kidney Research Fund.
John Sayer is funded by Kidney Research UK and the Northern Counties Kidney Research Fund. This research was made possible through access to the data and findings generated by the 100,000 Genomes Project. The 100,000 Genomes Project is managed by Genomics England Limited (a wholly owned company of the Department of Health and Social Care). The 100,000 Genomes Project is funded by the National Institute for Health Research and NHS England. The Wellcome Trust, Cancer Research UK and the Medical Research Council have also funded research infrastructure. The 100,000 Genomes Project uses data provided by patients and collected by the National Health Service as part of their care and support. See supplemental information for consortium details The DDD study presents independent research commissioned by the Health Innovation Challenge Fund [grant number HICF-1009-003], a parallel funding partnership between Wellcome and the Department of Health, and the Wellcome Sanger Institute [grant number WT098051]. The views expressed in this publication are those of the author(s) and not necessarily those of Wellcome or the Department of Health. The study has UK Research Ethics Committee approval (10/H0305/83, granted by the Cambridge South REC, and GEN/284/12 granted by the Republic of Ireland REC). The research team acknowledges the support of the National Institute for Health Research, through the Comprehensive Clinical Research Network. This study makes use of data generated by the DECIPHER community. A full list of centres who contributed to the generation of the data is available from https://deciphergenomics.org/about/stats and via email from contact@deciphergenomics.org. Funding for the DECIPHER project was provided by Wellcome. The DECIPHER community, carrying out the original analysis and collection of

For Review Only

the data, bears no responsibility for the further analysis or interpretation of the data.

Schematics were made using Biorender.com

Conflicts of Interest

The authors declare no conflict of interest.

For Review Only

References

1. Poretti A, Häusler M, von Moers A, et al. Ataxia, intellectual disability, and ocular apraxia with cerebellar cysts: a new disease? *Cerebellum (London, England)*. Feb 2014;13(1):79-88. doi:10.1007/s12311-013-0521-8
2. Aldinger KA, Mosca SJ, Tétreault M, et al. Mutations in LAMA1 cause cerebellar dysplasia and cysts with and without retinal dystrophy. *American journal of human genetics*. Aug 7 2014;95(2):227-34. doi:10.1016/j.ajhg.2014.07.007
3. Micalizzi A, Poretti A, Romani M, et al. Clinical, neuroradiological and molecular characterization of cerebellar dysplasia with cysts (Poretti-Boltshauser syndrome). *European journal of human genetics : EJHG*. Aug 2016;24(9):1262-7. doi:10.1038/ejhg.2016.19
4. Vilboux T, Malicdan MC, Chang YM, et al. Cystic cerebellar dysplasia and biallelic LAMA1 mutations: a lamininopathy associated with tics, obsessive compulsive traits and myopia due to cell adhesion and migration defects. *Journal of medical genetics*. May 2016;53(5):318-29. doi:10.1136/jmedgenet-2015-103416
5. Marlow E, Chan RVP, Oltra E, Rusu I, Gupta MP. Retinal Avascularity and Neovascularization Associated With LAMA1 (laminin1) Mutation in Poretti-Boltshauser Syndrome. *JAMA ophthalmology*. Jan 1 2018;136(1):96-97. doi:10.1001/jamaophthalmol.2017.5060
6. Masson R, Piretti E, Pellegrin S, et al. Early-onset head titubation in a child with Poretti-Boltshauser syndrome. *Neurology*. Apr 11 2017;88(15):1478-1479. doi:10.1212/wnl.0000000000003823
7. Banerjee A, Vyas S, Sankhyan N. Cerebellar Cysts and Dysplasias: More Diagnoses to Consider. *Pediatric neurology*. Sep 2019;98:91-92. doi:10.1016/j.pediatrneurol.2019.02.021
8. Bachmann-Gagescu R, Dempsey JC, Bulgheroni S, et al. Healthcare recommendations for Joubert syndrome. *American journal of medical genetics Part A*. Jan 2020;182(1):229-249. doi:10.1002/ajmg.a.61399
9. Joubert M, Eisenring JJ, Robb JP, Andermann F. Familial agenesis of the cerebellar vermis. A syndrome of episodic hyperpnea, abnormal eye movements, ataxia, and retardation. *Neurology*. Sep 1969;19(9):813-25. doi:10.1212/wnl.19.9.813
10. Maria BL, Hoang KB, Tusa RJ, et al. "Joubert syndrome" revisited: key ocular motor signs with magnetic resonance imaging correlation. *Journal of child neurology*. Oct 1997;12(7):423-30. doi:10.1177/088307389701200703
11. Kroes HY, van Zon PH, Fransen van de Putte D, et al. DNA analysis of AHI1, NPHP1 and CYCLIN D1 in Joubert syndrome patients from the Netherlands. *European journal of medical genetics*. Jan-Feb 2008;51(1):24-34. doi:10.1016/j.ejmg.2007.10.001
12. Nuovo S, Bacigalupo I, Ginevrino M, et al. Age and sex prevalence estimate of Joubert syndrome in Italy. *Neurology*. Feb 25 2020;94(8):e797-e801. doi:10.1212/wnl.0000000000008996
13. Parisi M, Glass I. Joubert Syndrome. In: Adam MP, Ardinger HH, Pagon RA, et al, eds. *GeneReviews(®)*. University of Washington, Seattle

Copyright © 1993-2020, University of Washington, Seattle. GeneReviews is a registered trademark of the University of Washington, Seattle. All rights reserved.; 1993.
14. Doherty D. Joubert syndrome: insights into brain development, cilium biology, and complex disease. *Seminars in pediatric neurology*. Sep 2009;16(3):143-54. doi:10.1016/j.spen.2009.06.002
15. Romani M, Micalizzi A, Valente EM. Joubert syndrome: congenital cerebellar ataxia with the molar tooth. *The Lancet Neurology*. Sep 2013;12(9):894-905. doi:10.1016/s1474-4422(13)70136-4
16. Bachmann-Gagescu R, Dempsey JC, Phelps IG, et al. Joubert syndrome: a model for untangling recessive disorders with extreme genetic heterogeneity. *Journal of medical genetics*. Aug 2015;52(8):514-22. doi:10.1136/jmedgenet-2015-103087
17. Shaheen R, Szymanska K, Basu B, et al. Characterizing the morbid genome of ciliopathies. *Genome biology*. Nov 28 2016;17(1):242. doi:10.1186/s13059-016-1099-5

For Review Only

18. Vilboux T, Doherty DA, Glass IA, et al. Molecular genetic findings and clinical correlations in 100 patients with Joubert syndrome and related disorders prospectively evaluated at a single center. *Genetics in medicine : official journal of the American College of Medical Genetics*. Aug 2017;19(8):875-882. doi:10.1038/gim.2016.204
19. Alkanderi S, Molinari E, Shaheen R, et al. ARL3 Mutations Cause Joubert Syndrome by Disrupting Ciliary Protein Composition. *American journal of human genetics*. Oct 4 2018;103(4):612-620. doi:10.1016/j.ajhg.2018.08.015
20. Latour BL, Van De Weghe JC, Rusterholz TD, et al. Dysfunction of the ciliary ARMC9/TOGARAM1 protein module causes Joubert syndrome. *The Journal of clinical investigation*. Aug 3 2020;130(8):4423-4439. doi:10.1172/jci131656
21. Prevalence and architecture of de novo mutations in developmental disorders. *Nature*. Feb 23 2017;542(7642):433-438. doi:10.1038/nature21062
22. Turro E, Astle WJ, Megy K, et al. Whole-genome sequencing of patients with rare diseases in a national health system. *Nature*. Jul 2020;583(7814):96-102. doi:10.1038/s41586-020-2434-2
23. Firth HV, Richards SM, Bevan AP, et al. DECIPHER: Database of Chromosomal Imbalance and Phenotype in Humans Using Ensembl Resources. *American journal of human genetics*. Apr 2009;84(4):524-33. doi:10.1016/j.ajhg.2009.03.010
24. Large-scale discovery of novel genetic causes of developmental disorders. *Nature*. Mar 12 2015;519(7542):223-8. doi:10.1038/nature14135
25. Schröder S, Li Y, Yigit G, et al. Heterozygous truncating variants in SUFU cause congenital ocular motor apraxia. *Genetics in medicine : official journal of the American College of Medical Genetics*. Feb 2021;23(2):341-351. doi:10.1038/s41436-020-00979-w
26. Maria BL, Quisling RG, Rosainz LC, et al. Molar tooth sign in Joubert syndrome: clinical, radiologic, and pathologic significance. *Journal of child neurology*. Jun 1999;14(6):368-76. doi:10.1177/088307389901400605
27. Epting D, Senaratne LDS, Ott E, et al. Loss of CBY1 results in a ciliopathy characterized by features of Joubert syndrome. *Human mutation*. Dec 2020;41(12):2179-2194. doi:10.1002/humu.24127
28. Barth PG, Majoie CB, Caan MW, et al. Pontine tegmental cap dysplasia: a novel brain malformation with a defect in axonal guidance. *Brain : a journal of neurology*. Sep 2007;130(Pt 9):2258-66. doi:10.1093/brain/awm188

For Review Only

Tables

Table 1. Clinical and neuro-imaging features of patients

Patient ID	NCL_Q73	GEL-01	GEL-02	NCL_Q98
Age	3 y	6 y 11 m	8 y	12 y
Sex	F	F	F	M
Ethnicity	White British	Caribbean / Gambian	White British	White British
Neurodevelopmental features	Moderate speech delay	Mild motor and speech delay	Motor delay	Motor and speech delay
Ataxia	Ataxia	Truncal and gait ataxia	Truncal Ataxia	Ataxia
Autistic features	Yes	No	No	Yes
Ocular motor apraxia	Yes	No	No	Yes
Myopia	Yes	Yes	Yes	Yes
Cerebellar dysplasia	Yes	Yes	Yes	Yes
Cerebellar cysts	Yes	Yes	Yes	Yes
Molar tooth sign	No	No	No	No

For Review Only

Additional brain MRI features	Splayed superior cerebellar peduncles	Abnormal shape of the fourth ventricle	Abnormal rhomboid shape of fourth ventricle in axial and sagittal plane, Splayed superior cerebellar peduncles	Abnormal rhomboid shape of fourth ventricle in axial and sagittal plane
Other		Total Anomalous Pulmonary Venous Drainage	Branchial cyst	

For Review Only

Table 2. Molecular genetic investigations

Patient ID	Prior molecular genetic tests	Molecular tests applied in this study	*LAMA1* alleles identified (NM_005559)	Pathogenicity (MutationTaster) & gnomAD allele frequency (Allele counts/Homoz./Total alleles)
NCL_Q73	29 genes panel for Joubert syndrome: negative	Duo whole exome sequencing (proband and mother)	1. c.494delT, p.(Ile165fs*16) (Het);<br>2. c.3053delC, p.(Pro1018fs*6) (Het)	1. Disease causing; not in gnomAD<br>2. Disease causing; not in gnomAD
GEL-01	Whole Genome Sequencing (Genomics England): unsolved	Re-evaluation of WGS data	1. c.281A>G, p.(Gln94Arg)# (Het);<br>2. c.3397C>T, p.(Arg1133*) (Het)	1. Disease causing;not in gnomAD<br>2. Disease causing; 5/0/250388
GEL-02	Hereditary ataxia gene panel: negative, Whole Genome Sequencing (Genomics England): unsolved	Re-evaluation of WGS data	1. c.2344C>T, p.(Arg782*) (Het);<br>2. c.2962delT, p.(Tyr988Thrfs*3) (Het)	1. Disease causing, ClinVar: Pathogenic;11/0/272248<br>2. Disease causing; not in gnomAD
NCL_Q98	18 genes panel for Joubert syndrome: negative<br>Microarray analysis Mat inherited variant 4q28.1, likely benign	DDD study recruitment Array CGH and WES	1. c.1281C>A, p.(Cys427*) (Het);<br>2. c.2344C>T, p.(Arg782*) (Het)	1. Disease causing; not in gnomAD<br>2. Disease causing, ClinVar: Pathogenic; 11/0/272248

MutationTaster: "Disease Causing"; SIFT: "deleterious"; PolyPhen: "probably damaging"; CADD score 26.4; REVEL score 0.923. Gln94 is highly conserved and lies within the Laminin alpha-1 LN domain (Figure S6)

For Review Only

Figure legends

Figure 1. Diagnosis of Poretti-Boltshauser syndrome in four British families

(A) Brain MRI findings in proband NCL_Q73 demonstrate (i) axial view at the level of the upper vermis showing multiple cysts (circle) and elongated splayed superior cerebellar peduncles (arrow); (ii) axial view demonstrating an abnormal shaped enlarged fourth ventricle and multiple cysts in the peripheral parts of the hemispheres; (iii) coronal view showing multiple cysts in the upper parts of the cerebellum and marked dysplasia; (iv) sagittal view demonstrating an enlarged quadrangular fourth ventricle (asterisk) and small cysts in the anterior vermis.(B) *LAMA1* molecular genetic results in index case NCL_Q73 and (C) three additional cases identified in this study. Probands are identified by arrows. Shaded symbols show affected cases, males squares, females circles.

For Review Only

Figure 2. Genetic diagnosis of Poretti-Boltshauser syndrome and Joubert syndrome in patients with developmental disorders and intellectual disabilities

(A) Number of cases solved for either *LAMA1* (blue) or Joubert syndrome genes (greyscale) among 8459 probands recruited with HPO terms “intellectual disability” and/or “developmental delay” in Genomics England 100,000 Genomes Project.

(B) Number of cases solved for either *LAMA1* (blue) or Joubert syndrome genes (greyscale) among ~14,000 probands recruited in the UK Deciphering Developmental Disorders study and with phenotypical data indicating either intellectual disability or developmental delay.

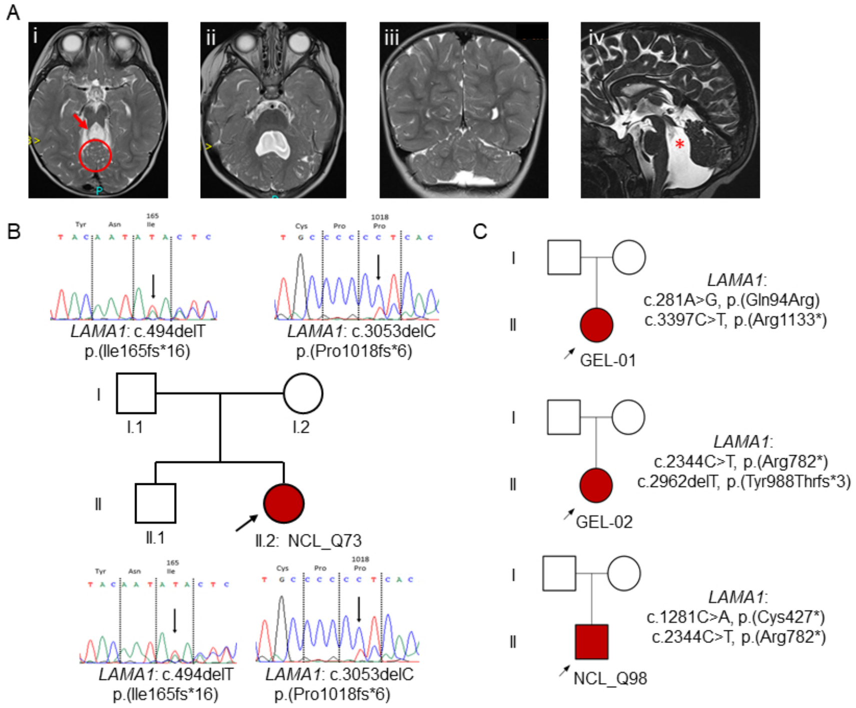

Figure 1. Diagnosis of Poretti-Boltshauser syndrome in four British families
(A) Brain MRI findings in proband NCL_Q73 demonstrate (i) axial view at the level of the upper vermis showing multiple cysts (circle) and elongated splayed superior cerebellar peduncles (arrow); (ii) axial view demonstrating an abnormal shaped enlarged fourth ventricle and multiple cysts in the peripheral parts of the hemispheres; (iii) coronal view showing multiple cysts in the upper parts of the cerebellum and marked dysplasia; (iv) sagittal view demonstrating an enlarged quadrangular fourth ventricle (asterisk) and small cysts in the anterior vermis.(B) LAMA1 molecular genetic results in index case NCL_Q73 and (C) three additional cases identified in this study. Probands are identified by arrows. Shaded symbols show affected cases, males squares, females circles.

254x190mm (96 x 96 DPI)

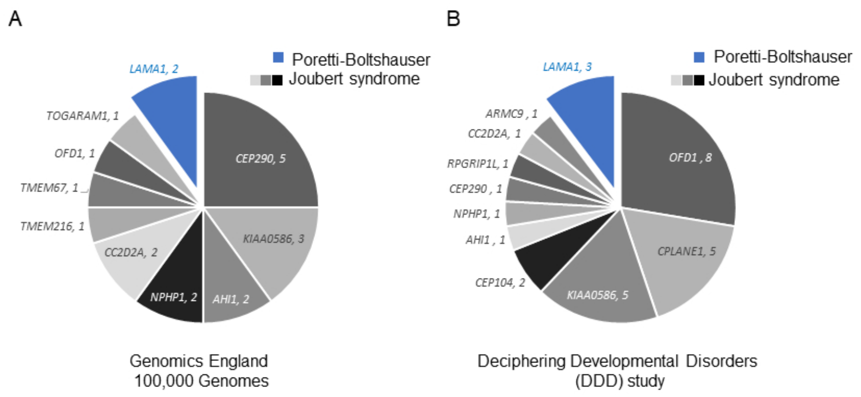

Figure 2. Genetic diagnosis of Poretti-Boltshauser syndrome and Joubert syndrome in patients with developmental disorders and intellectual disabilities
(A) Number of cases solved for either LAMA1 (blue) or Joubert syndrome genes (greyscale) among 8459 probands recruited with HPO terms "intellectual disability" and/or "developmental delay" in Genomics England 100,000 Genomes Project.
(B) Number of cases solved for either LAMA1 (blue) or Joubert syndrome genes (greyscale) among ~14,000 probands recruited in the UK Deciphering Developmental Disorders study and with phenotypical data indicating either intellectual disability or developmental delay.

254x190mm (96 x 96 DPI)

Supplementary Information

Identification of *LAMA1* mutations ends diagnostic odyssey and has prognostic implications for patients with presumed Joubert syndrome

For Review Only

Appendix: Genomics England Research Consortium
Supplementary Tables S1-S4
Supplementary Figures S1-S10
Supplementary References

Appendix: Genomics England Research Consortium

Ambrose, J. C. [1] ; Arumugam, P.[1] ; Bleda, M. [1] ; Boardman-Pretty, F. [1,2] ; Boustred, C. R. [1] ; Brittain, H.[1] ; Caulfield, M. J.[1,2] ; Chan, G. C. [1] ; Fowler, T. [1] ; Giess A. [1] ; Hamblin, A.[1] ; Henderson, S.[1,2] ; Hubbard, T. J. P. [1] ; Jackson, R. [1] ; Jones, L. J. [1,2] ; Kasperaviciute, D. [1,2] ; Kayikci, M. [1] ; Kousathanas, A. [1] ; Lahnstein, L. [1] ; Leigh, S. E. A. [1] ; Leong, I. U. S. [1] ; Lopez, F. J. [1] ; Maleady-Crowe, F. [1] ; Moutsianas, L. [1,2] ; Mueller, M. [1,2] ; Murugaesu, N. [1] ; Need, A. C. [1,2] ; O'Donovan P. [1] ; Odhams, C. A. [1] ; Patch, C. [1,2] ; Perez-Gil, D. [1] ; Pereira, M. B. [1] ; Pullinger, J. [1] ; Rahim, T. [1] ; Rendon, A. [1] ; Rogers, T. [1] ; Savage, K. [1] ; Sawant, K. [1] ; Scott, R. H. [1] ; Siddiq, A. [1] ; Sieghart, A. [1] ; Smith, S. C. [1] ; Sosinsky, A. [1,2] ; Stuckey, A. [1] ; Tanguy M. [1] ; Thomas, E. R. A. [1,2] ; Thompson, S. R. [1] ; Tucci, A. [1,2] ; Walsh, E. [1] ; Welland, M. J. [1] ; Williams, E. [1] ; Witkowska, K. [1,2] ; Wood, S. M. [1,2].

[1] Genomics England, London, UK

[2] William Harvey Research Institute, Queen Mary University of London, London, EC1M 6BQ, UK

For Review Only

Table S1. Comparison of clinical features of Joubert syndrome and Poretti-Boltshauser syndrome

	Joubert Syndrome	Poretti-Boltshauser Syndrome
**Infancy**	Hypotonia<br>Delay in motor and speech development<br>Ocular motor apraxia<br>Intellectual disability<br>Ataxia<br>Abnormal breathing pattern (not consistent)<br>Facial dysmorphic features, including frontal bossing, high-arched eyebrows, epicanthic folds, anteverted nostrils, long philtrum, open mouth, protruded tongue (not consistent)	Hypotonia<br>Delay in motor and speech development<br>Ocular motor apraxia<br>Intellectual disability<br>Ataxia
**Additional eye phenotypes**	Retinal dystrophy<br>Nystagmus<br>Strabismus<br>Ptosis<br>Ocular coloboma	Retinal dystrophy<br>Nystagmus<br>Strabismus<br>Retinal atrophy<br>Increased retinal pigment<br>Macular heterotopia<br>High myopia
**Other phenotypes**	Nephronophthisis<br>Hepatic fibrosis<br>Postaxial polydactyly<br>Bone shortening, skeletal dysplasia<br>Oral hamartomas<br>Congenital heart defects	

Red are shared features, black may be distinguishing features, but are not consistent or age dependent

For Review Only

Table S2. Comparison of brain MRI features of Joubert syndrome and Poretti-Boltshauser syndrome (see Figures S1-S4)

Joubert Syndrome	Poretti-Boltshauser Syndrome
Molar Tooth Sign (resulting from deep interpeduncular fossa and elongated thickened superior cerebellar peduncles)<br><br>Cerebellar vermis hypoplasia / dysplasia<br>Upper vermis: folial dysplasia<br>Abnormal shape 4th ventricle, dislocated fastigium<br><br>(No cerebellar cysts)	Cerebellar dysplasia<br>Cerebellar cysts<br>Abnormally shaped 4th ventricle (in axial and sagittal plane)<br>Superior cerebellar peduncles splayed (coronal)

view Only

Table S3. Known genetic causes of Joubert syndrome

Phenotype	Gene	Gene MIM number
**Joubert syndrome 1**	*INPP5E*	613037
**Joubert syndrome 2**	*TMEM216*	613277
**Joubert syndrome 3**	*AHI1*	608894
**Joubert syndrome 4**	*NPHP1*	607100
**Joubert syndrome 5**	*CEP290*	610142
**Joubert syndrome 6**	*TMEM67*	609884
**Joubert syndrome 7**	*RPGRIP1L*	610937
**Joubert syndrome 8**	*ARL13B*	608922
**Joubert syndrome 9**	*CC2D2A*	612013
**Joubert syndrome 10**	*OFD1*	300170
**Joubert syndrome 11**	*TTC21B*	612014
**Joubert syndrome 12**	*KIF7*	611254
**Joubert syndrome 13**	*TECT1/TCTN1*	609863
**Joubert syndrome 14**	*TMEM237*	614423
**Joubert syndrome 15**	*CEP41*	610523
**Joubert syndrome 16**	*TMEM138*	614459
**Joubert syndrome 17**	*CPLANE1/C5orf42*	614571
**Joubert syndrome 18**	*TCTN3*	613847
**Joubert syndrome 19**	*ZNF423*	604557
**Joubert syndrome 20**	*TMEM231*	614949

For Review Only

**Joubert syndrome 21**	*CSPP1*	611654
**Joubert syndrome 22**	*PDE6D*	602676
**Joubert syndrome 23**	*KIAA0586*	610178
**Joubert syndrome 24**	*TCTN2*	613846
**Joubert syndrome 25**	*CEP104*	616690
**Joubert syndrome 26**	*KATNIP*	616650
**Joubert syndrome 27**	*B9D1*	614144
**Joubert syndrome 28**	*MKS1*	609883
**?Joubert syndrome 29**	*TMEM107*	616183
**Joubert syndrome 30**	*ARMC9*	617612
**Joubert syndrome 31**	*CEP120*	613446
**Joubert syndrome 32**	*SUFU*	607035
**Joubert syndrome 33**	*PIBF1*	607532
**Joubert syndrome 34**	*B9D2*	614175
**Joubert syndrome 35**	*ARL3*	604695
**Joubert syndrome 36**	*FAM149B1*	618413
**Joubert syndrome 37**	*TOGARAM1*	617618
**N/A**	*CBY1*	N/A[1]

For Review Only

Table S4. Summary of reported Poretti-Boltshauser patients

Patient ID from Publication	*LAMA1* variants (NM_005559)	Age of MRI / Diagnosis	Educational Achievement (age)	REF
CA0035	c.588+2T>G (Hom)	36 months at diagnosis	Not known (36 m)	2
UW154-3	c.6345+3G>C (Het);<br>Del exons 4-11 (Het)	36 months at diagnosis	Not known (36 m)	2
UW162-3	c.7965-15_79653del (Het);<br>c.2988_2989delA, p.(Pro996Hisfs28*) (Het)	9 months at diagnosis	Not known (25 months)	2
UW160-3	c.6701delC, p.(Pro2334Leufs9*) (Het);<br>c.8557-1G>C (Het);<br>c.768+1G>A (Het)	Unknown	UW 160-3 at 29 years - Normal IQ College graduate, lives independently	2
UW160-4	c.6701delC, p.(Pro2334Leufs9*) (Het);<br>c.8557-1G>C (Het);<br>c.768+1G>A (Het)	Unknown	UW 160-4 At 23 years autism spectrum disorder (Asperger), lives with parents	2
UW163-3 (Family 1, Patient 1)	c.2816_2817delAT, p.(Tyr939Leu27*) (Het);<br>c.555T>G, p.(Tyr185*) (Het)	2 years 1 month	Not known (26 years)	2,3
UW163-4 (Family 1, Patient 2)	c.2816_2817delAT, p.(Tyr939Leu27*) (Het);<br>c.555T>G, p.(Tyr185*) (Het)	5 months	Not known (21 years)	2,3
Family 2, Patient 3	c.2160T>A, p.(Cys720*) (Het);<br>c.5985_5991del, p.(Ile1996Glufs*7) (Het)	8 years 6 months	Not known (8 years 6 months)	3
Patient 1	c.664C>T, p.(Arg222*) (Het);<br>c.2331C>G, p.(Tyr777*) (Het)	<5 years	Not known (8 years)	4
Patient 2	c.664C>T, p.(Arg222*) (Het);<br>c.2331C>G, p.(Tyr777*) (Het)	<5 years	Not known (8 years)	4

Patient 1	c.4702_4703del; p.(Leu1568Glyfs*2) (Hom)	2 years 6 months	Not known (2 years 6 months)	5
Patient 1	c.8192C>A, p.(Ser2731*) (Hom)	7 years	Not known (7 years)	6
Patient 2	c.8192C>A, p.(Ser2731*) (Hom)	7 years	Not known (7 years(	6
Patient 1	c. 8556+1G>A (Hom)	4 months	IQ 70 (12 years 4 months)	7
Patient 2	c.2935delA, p.(Arg979Glyfs*45) (Hom)	5 months	IQ 60 (7 years 4 months)	7
Patient 3	c.4676delA, p.(Glu1559Glyfs*3) (Het) c.7180C>T; p.(Arg2394*) (Het)	3 months	Developmental delay (6 years 6 months)	7
Patient 4	c.2935delA, p.(Arg979Glyfs*45) (Hom)	3-6 months	Developmental delay (14 years)	7
Patient 5	c.2935delA, p.(Arg979Glyfs*45) (Hom)	3-6 months	Developmental delay (8 years 9 months)	7
Patient 6	c.2935delA, p.(Arg979Glyfs*45) (Hom)	3-6 months	Developmental delay (7 years 6 months)	7
Patient 7	c.2935delA, p.(Arg979Glyfs*45) (Hom)	3-6 months	Developmental delay (11 years)	7
Patient 8	c.1774_1775insTTCATAAT, p.(Ser592Phefs*9) (Het); c.6348dupT, p.(Lys2117*) (Het)	2 months	IQ 99 (8 years 9 months)	7
Patient 9	c.2935delA, p.(Arg979Glyfs*45) (Hom)	4 months	Developmental delay (4 years)	7
Patient 10	c.470C>G, p.(Ser157*) (Het); g.6999443_6999910del, p.(Phe1462Lysfs*1) (Het)	3-4 months	Developmental delay (5 years 6 months)	7
Patient 11	c.2935delA, p.(Arg979Glyfs*45) (Hom)	3-4 months	Developmental delay (3 years 6 months)	7

Patient 12	c.4663+1G>C (Het);<br>c.1404_1405delA, p.(Gly469Alafs*5) (Het)	3-4 months	Developmental delay (12 years)	[7]
Patient 13	c.2935delA, p.(Arg979Glyfs*45) (Het);<br>c.2616delG; p.(Lys872Asnfs*23) (Het)	3-4 months	Developmental delay (2 years)	[7]
Patient 14	c.8761C>T, p.(Arg2921*) (Het);<br>g.6942238_6943401del, p.(Val2929Serfs?) (Het)	2-3 months	Developmental delay (1 years 6 months)	[7,8]
Patient 15	c.164A>T, p.(His55Leu) (Het);<br>c.2108C>T, p.(Ala703Val) (Het)	2-3 months	IQ 118 (9 years 6 months)	[7]
Patient 16	c.3919C>T; p.(Arg1307*) (Hom)	4-5 months	Developmental delay (7 years 6 months) Attended higher education. Running own business (22 years)	[7]
Patient 17	c.2935delA, p.(Arg979Glufs*45) (Hom)	4-5 months	Developmental delay (16 years)	[7]
DDD Study 323516	c.362_363del, p.(Tyr121Cysfs*4) (Hom)	10 years	Normal intelligence (10 years)	[9]
DDD Study 285238	c,5213del, p.(Lys1738Ser*9) (Het);<br>c.891C>A, p.(Cys297*) (Het)	N/A	N/A	[9]
Patient 1	c.2344C>T, p. (Arg782*) (Het); c.5512C>T, p.(Gln1838*) (Het)	3 years	N/A	[10]
Patient 1	c.7160G>T; p.(Trp2387*) (Hom)	2 years 6 months	Developmental delay (2 years 6 months)	[11]
Patient 1	c.4171_4172delAG, p.(Arg1391fs*19) (Hom)	2 years (OMA), 18 years brain MRI	Fluent in 3 languages, normal intelligence, studying within higher education college (18 years)	(Personal communication)

,

Figure S1. Normal brain MRI imaging

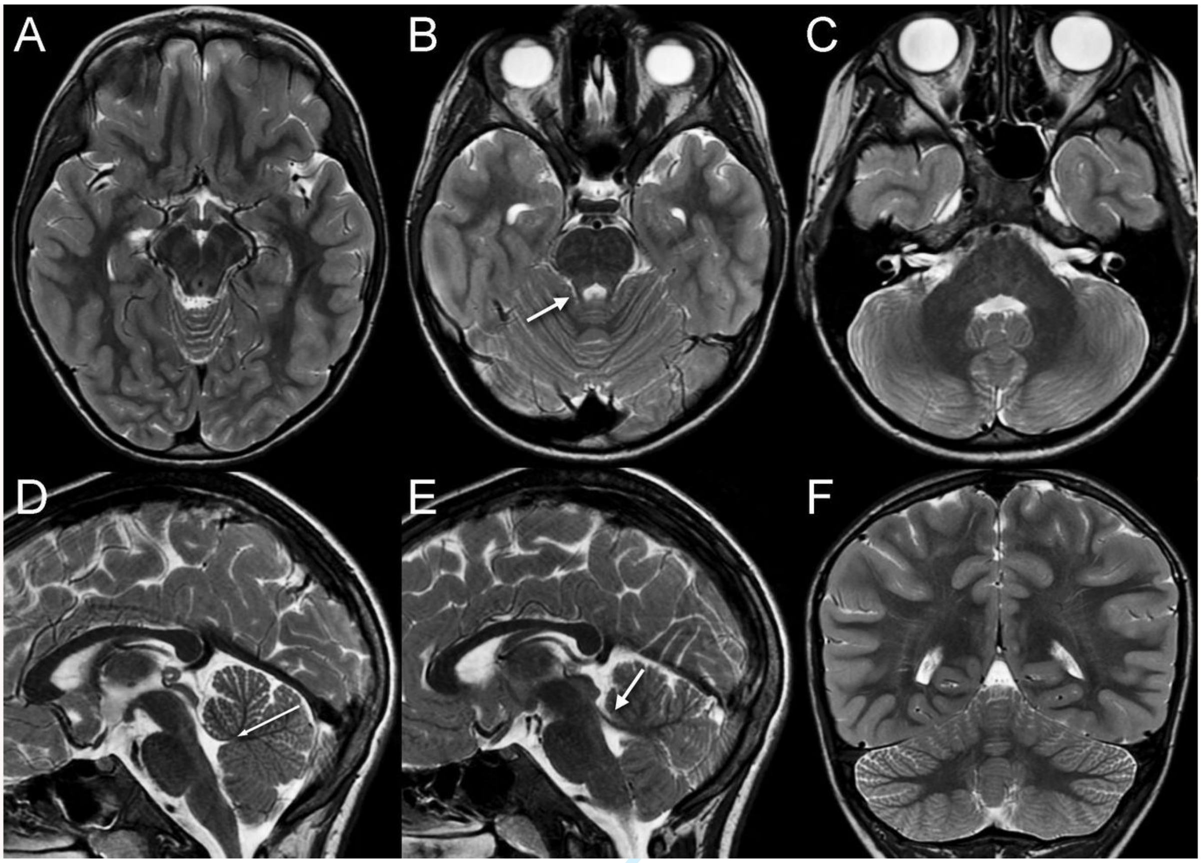

All images are T2w MRI. (A) Axial view at level of upper vermis showing regular folia. (B) Axial view showing normal dimensions of superior cerebellar peduncles (arrow). (C) Axial view at the level of normal shaped fourth ventricle. (D) Mid-sagittal view showing normal tent-like shape of fourth ventricle and a normal position of the fastigium (arrow). (E) Para-sagittal view showing normal sized and angulated superior cerebellar peduncle (arrow). (F) Coronal views showing normal white matter arborisation and regular vermis.

Figure S2. Typical brain MRI imaging in Poretti-Boltshauser syndrome (*LAMA1* mutations)

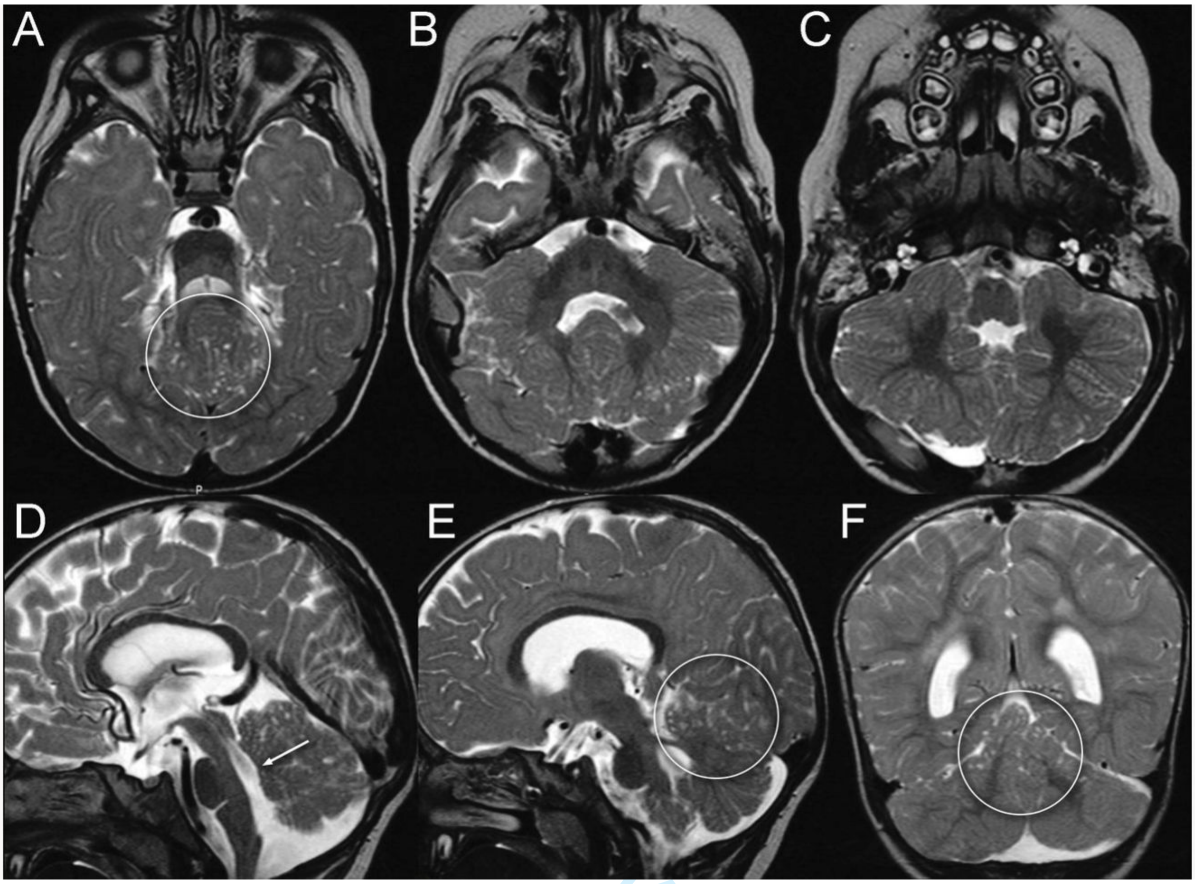

All images are T2w MRI. (A) Axial view at the level of the upper vermis showing no foliation but multiple small cysts (within circle). (B) Axial view demonstrating abnormal shape of fourth ventricle, abnormal white matter arborisation, and multiple small cysts. (C) Axial view at the level of the medulla oblongata showing marked dysplasia, i.e., irregular folial and white matter anatomy. (D) Mid-sagittal view demonstrating rhomboid-shaped fourth ventricle (arrow), absent vermis foliation, and multiple small cysts in the upper vermis. (E) Para-sagittal view: multiple small cysts in upper vermis (within circle). (F) Coronal views showing multiple cysts (within circle) and irregular architecture (compare with Figure S1 (F)).

Figure S3. Typical brain MRI imaging in Joubert syndrome (*CEP290* mutations)

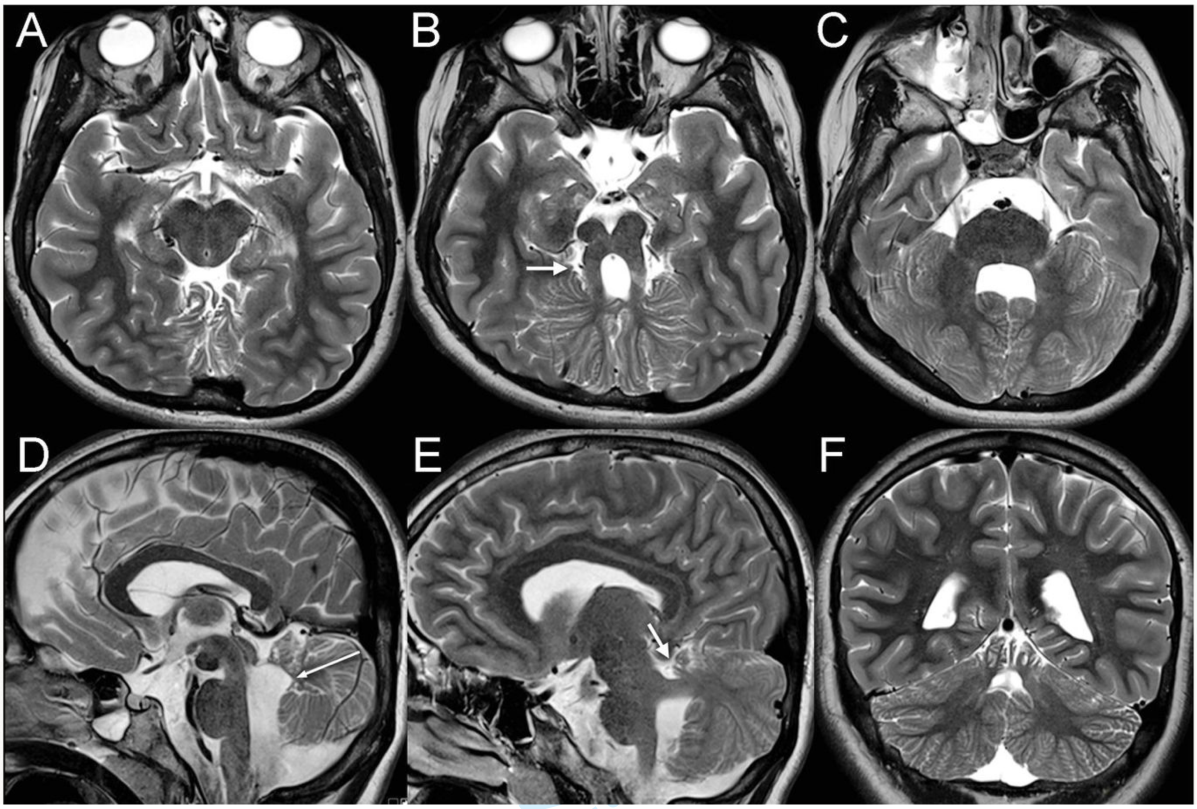

All images are T2w MRI. (A) Axial view at the level of the upper vermis showing irregular arrangement of folia. (B) Axial view at the level of the markedly thickened and elongated superior cerebellar peduncles (arrow) resulting in a “Molar Tooth Sign”. The crown of the tooth is asymmetric, a common finding. (C) Axial view at the level of the fourth ventricle showing a “bat-wing” shape. Posteriorly there is a small cleft between the cerebellar hemispheres due to vermis hypoplasia. (D) Mid-sagittal view showing abnormal shape of the fourth ventricle (compare with normal MRI (Figure S1) and cranial dislocation of fastigium (arrow). (E) Para-sagittal views demonstrating markedly thickened superior cerebellar peduncle with a horizontal course (arrow). (F) Coronal view showing irregular upper vermis with clefts.

Figure S4. Typical brain MRI imaging in Joubert syndrome (*INPP5E mutations*)

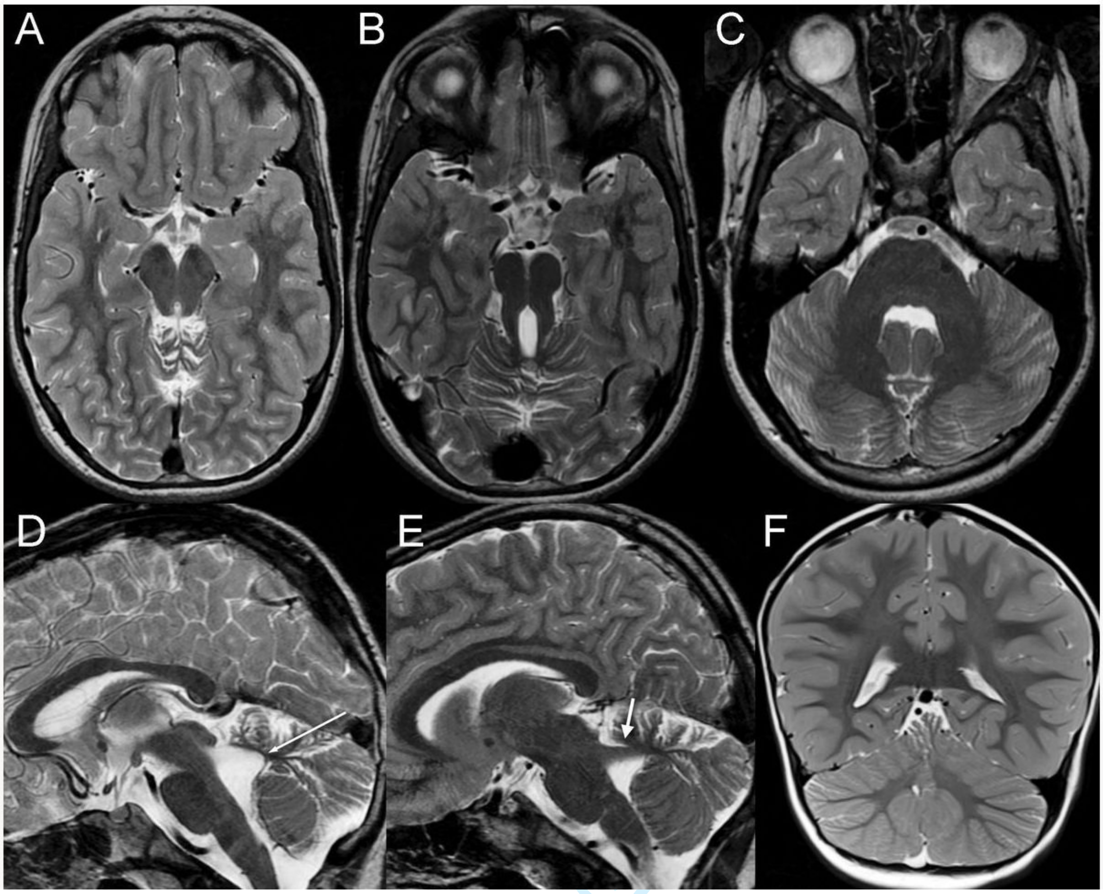

All images are T2w MRI. (A) Axial view at the level of the upper vermis showing irregular arrangement of folia. (B) Axial view at the level of the markedly thickened and elongated superior cerebellar peduncles (arrow) resulting in a “Molar Tooth Sign”. (C) Axial view at the level of the fourth ventricle showing a “bat-wing” shape. (D) Mid-sagittal view showing abnormal shape of the fourth ventricle (compare with normal MRI (Figure S1) and cranial dislocation of fastigium (arrow). (E) Para-sagittal views demonstrating markedly thickened superior cerebellar peduncle with a horizontal course (arrow). (F) Coronal view showing irregular upper vermis with clefts.

Figure S5. Sanger chromatogram confirmation of pathogenic *LAMA1* variants

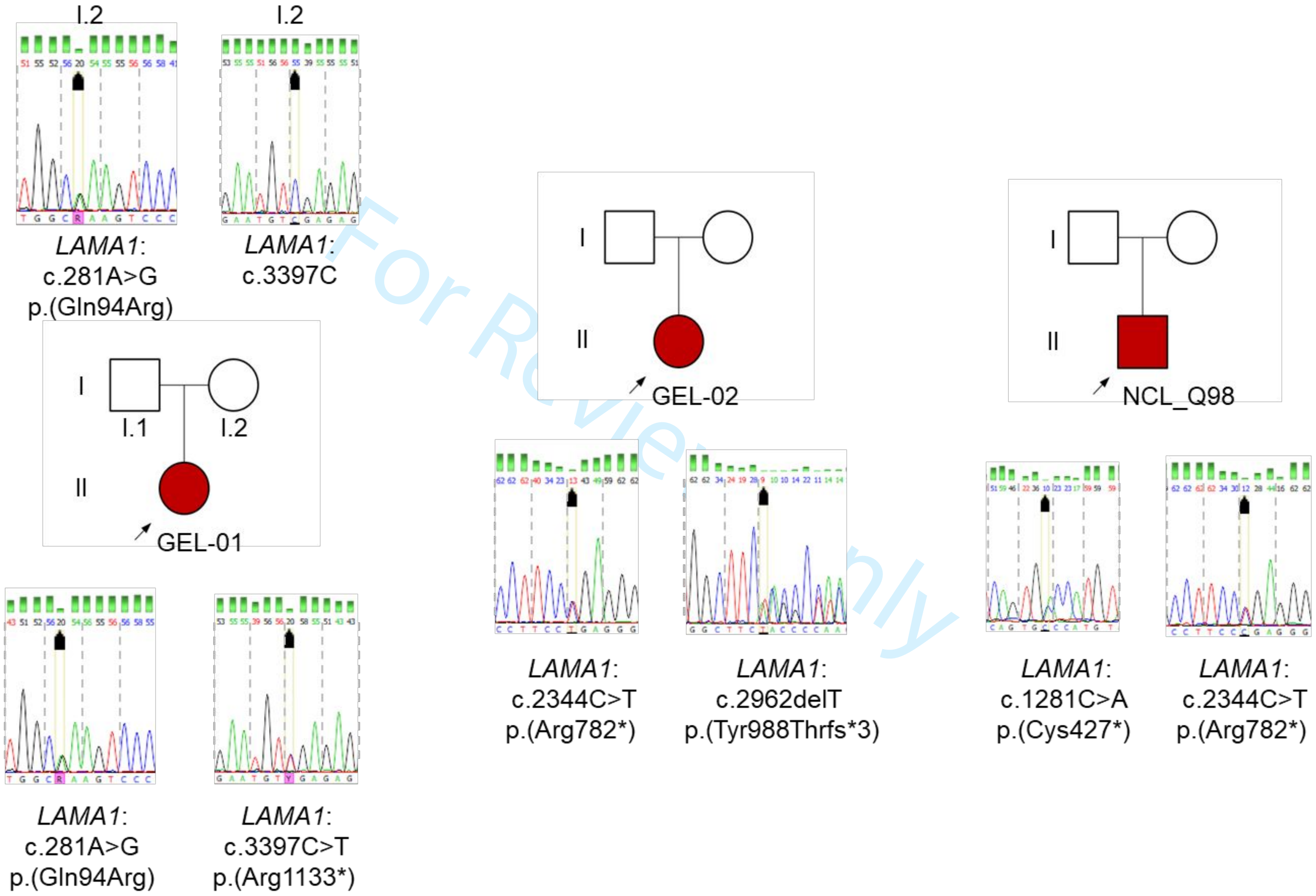

For Review Only

Figure S6. In silico modelling of the *LAMA1* missense allele Gln94Arg

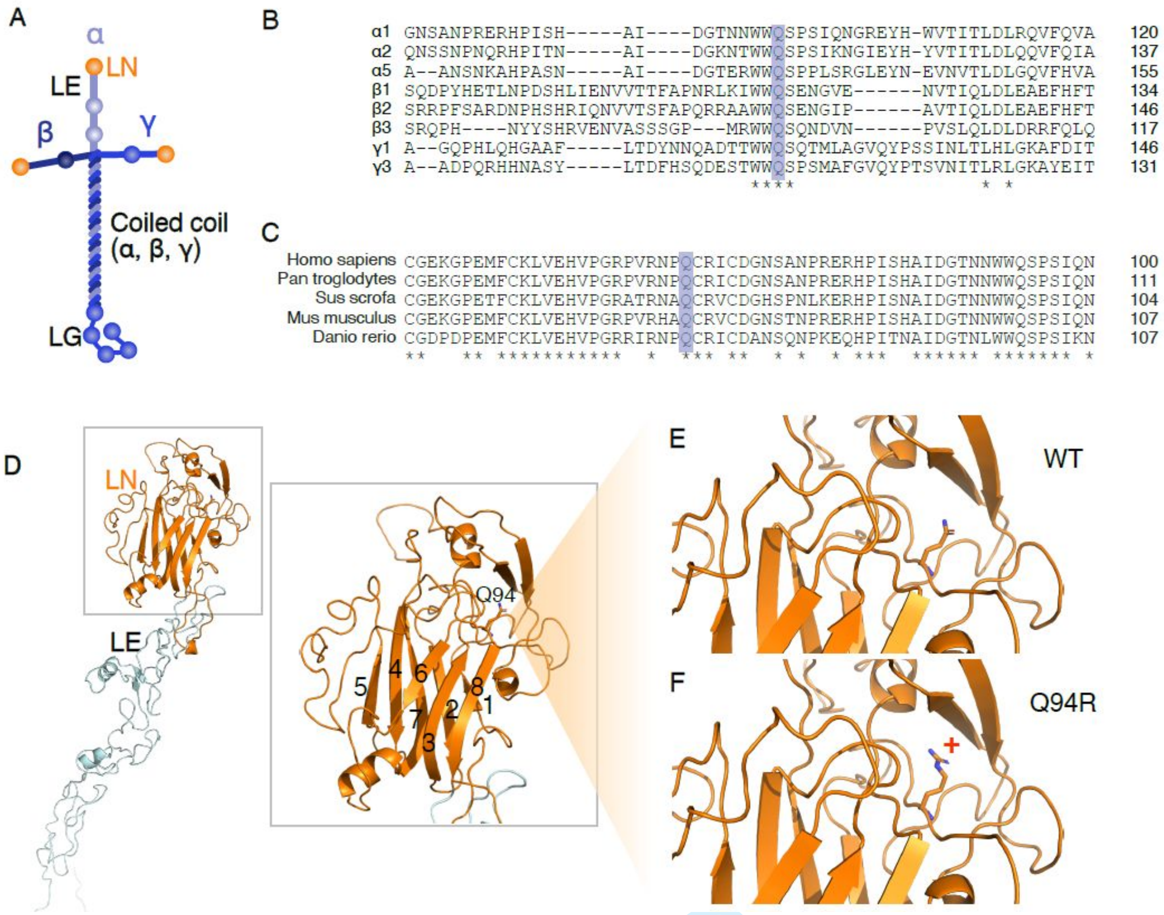

The laminin α1 Q94 residue is highly conserved and falls in the LN domain. (A) Schematic diagram of the asymmetric cross-shaped laminin molecule, consisting of three short arms α (pale blue), β (dark blue), and γ (mid-blue) and a single long arm: a coiled coil of α,β,γ. LN, N-terminal domain (orange); LE, epithelial growth factor (EGF)-like repeats interrupted by globular domains (blue); LG, C-terminal globular domains. (B) Multiple sequence alignment of human laminin isoforms containing a single LN domain. The Q94 residue is highlighted in blue. *, Conserved residue. C, Multiple sequence alignment of laminin α1 in vertebrates. The Q94 residue is highlighted in blue. *, Conserved residue. (D) Homology model of human laminin α1 based upon structural homology to mouse laminin β1 (PDB 4AQS). A zoomed in panel of the LN domain (orange) shows the 8 βstrands of the “jelly roll” fold (numbered) and the position of the Q94 residue. (E) Zoomed in image of the Q94 residue in the wild-type (WT) structure. (F) *In silico* mutagenesis of laminin α1 Q94R, resulting in the presence of a positively charged arginine residue in the LN domain.

Figure S7. Brain MRI of case GEL-01

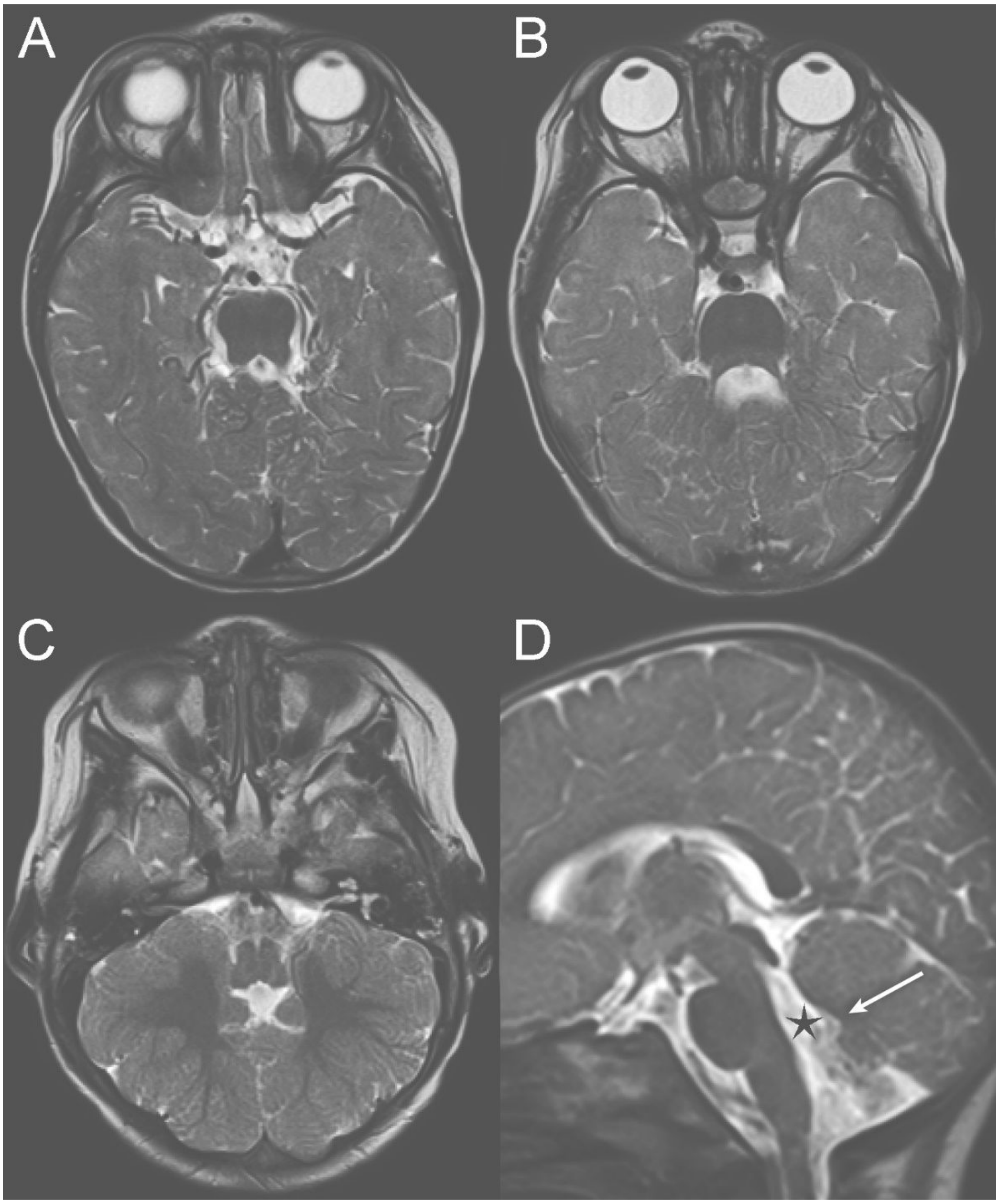

(A) Axial view at the level of the upper vermis showing dysplastic vermis and a few small cysts. (B) Axial view at the level of the enlarged and abnormally shaped fourth ventricle and splayed superior cerebellar peduncles. (C) Axial cut at the level of the medulla oblongata demonstrating dysplasia, i.e. abnormal white matter and folial anatomy. (D) Sagittal section demonstrating an abnormally shaped fourth ventricle (asterisk), the fastigium is caudally dislocated (arrow).

Figure S8. Brain MRI of case GEL-02

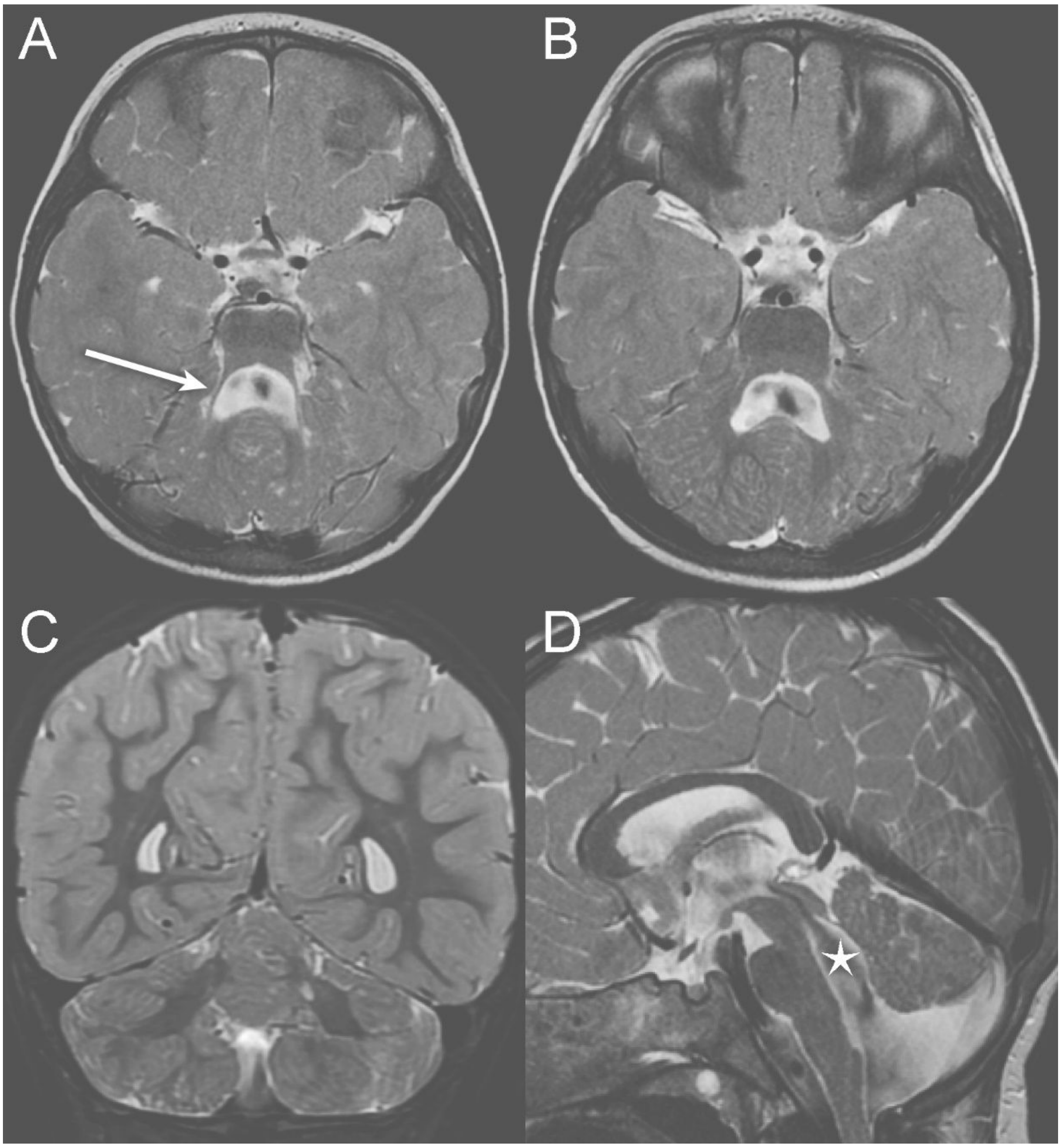

(A) Axial view at the level of the upper vermis showing thin splayed superior cerebellar peduncles and multiple small cysts behind. (B) Axial view demonstrating an abnormal shape and size of the fourth ventricle. (C) Coronal view showing disorganized white matter arborisation and foliation. (D) Sagittal view showing a rhomboid shaped fourth ventricle (asterisk), the vermis is not lobulated. (Flowing cerebro-spinal fluid leads to black signal in the aqueduct and fourth ventricle)

Figure S9. Brain MRI of case NCL_Q98

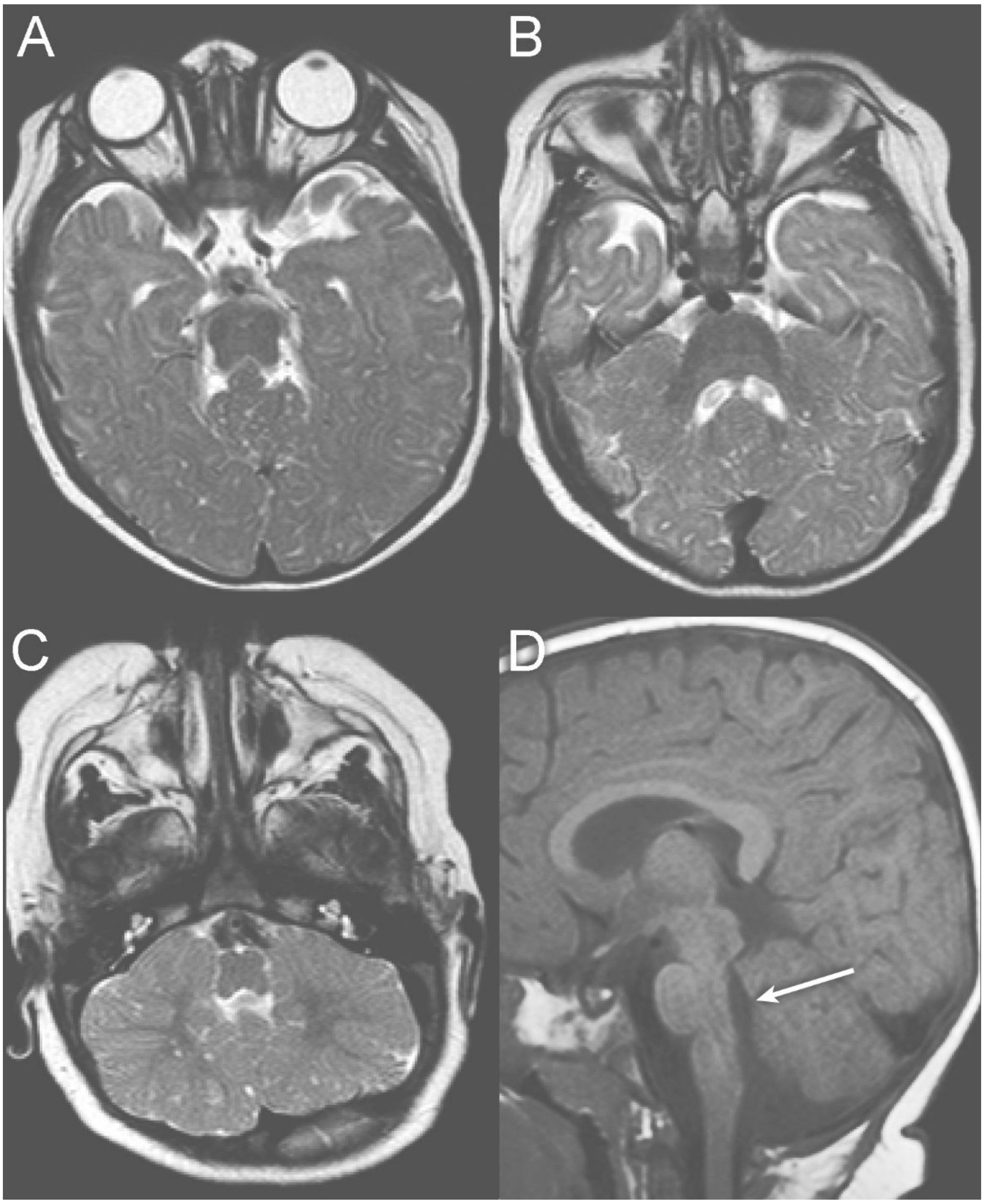

(A) Axial view at the level of the upper vermis (projected behind the brainstem) with multiple small cysts. (The cerebral white matter is not yet myelinated, appropriate for young age). (B) Axial cut at the level of the abnormal wide fourth ventricle, with multiple cysts in the vermis. (C) Axial section at the level of the medulla oblongata demonstrating dysplasia and small cysts. (D) Sagittal (T1) view showing a small flat ventricle (arrow).

Figure S10. Infographic comparing Poretti-Boltshauser syndrome and Joubert syndrome

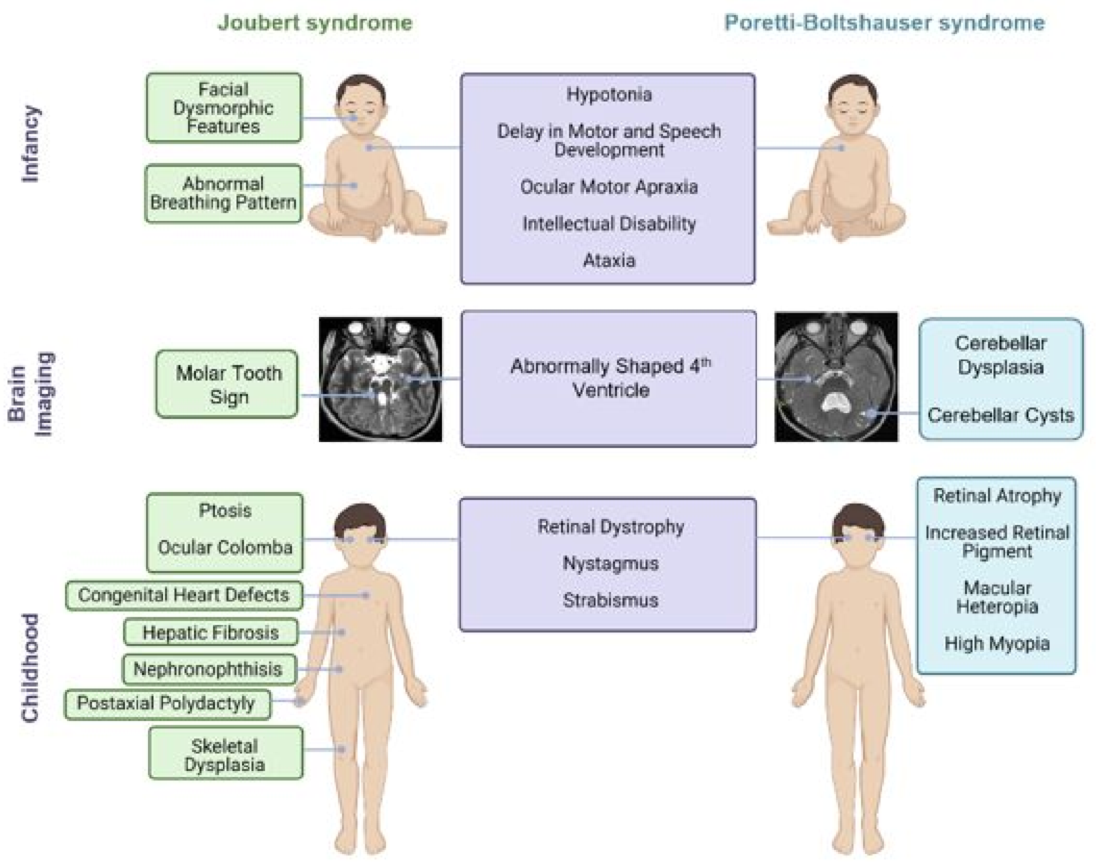

Supplementary References

1. Epting D, Senaratne LDS, Ott E *et al*: Loss of CBY1 results in a ciliopathy characterized by features of Joubert syndrome. *Human mutation* 2020; **41:** 2179-2194.

2. Aldinger KA, Mosca SJ, Tétreault M *et al*: Mutations in LAMA1 cause cerebellar dysplasia and cysts with and without retinal dystrophy. *American journal of human genetics* 2014; **95:** 227-234.

3. Vilboux T, Malicdan MC, Chang YM *et al*: Cystic cerebellar dysplasia and biallelic LAMA1 mutations: a lamininopathy associated with tics, obsessive compulsive traits and myopia due to cell adhesion and migration defects. *Journal of medical genetics* 2016; **53:** 318-329.

4. Marlow E, Chan RVP, Oltra E, Rusu I, Gupta MP: Retinal Avascularity and Neovascularization Associated With LAMA1 (laminin1) Mutation in Poretti-Boltshauser Syndrome. *JAMA ophthalmology* 2018; **136:** 96-97.

5. Banerjee A, Vyas S, Sankhyan N: Cerebellar Cysts and Dysplasias: More Diagnoses to Consider. *Pediatric neurology* 2019; **98:** 91-92.

6. Elmas M, Gogus B, Solak M: Understanding What You Have Found: A Family With a Mutation in the LAMA1 Gene With Literature Review. *Clinical medicine insights Case reports* 2020; **13:** 1179547620948666.

7. Micalizzi A, Poretti A, Romani M *et al*: Clinical, neuroradiological and molecular characterization of cerebellar dysplasia with cysts (Poretti-Boltshauser syndrome). *European journal of human genetics : EJHG* 2016; **24:** 1262-1267.

8. Masson R, Piretti E, Pellegrin S *et al*: Early-onset head titubation in a child with Poretti-Boltshauser syndrome. *Neurology* 2017; **88:** 1478-1479.

9. Firth HV, Richards SM, Bevan AP *et al*: DECIPHER: Database of Chromosomal Imbalance and Phenotype in Humans Using Ensembl Resources. *American journal of human genetics* 2009; **84:** 524-533.

10. Cai CX, Go M, Kelly MP, Holgado S, Toth CA: OCULAR MANIFESTATIONS OF PORETTI-BOLTSHAUSER SYNDROME: FINDINGS FROM MULTIMODAL IMAGING AND ELECTROPHYSIOLOGY. *Retinal cases & brief reports* 2020.

11. Alahmadi AS, Badawi AH, Magliyah MS, Albakri A, Schatz P: Poretti-Boltshauser syndrome: a rare differential diagnosis to consider in pediatric high myopia with retinal degeneration. *Ophthalmic genetics* 2021; **42:** 96-98.

For Review Only
