# Supplementary material for: Identification of LAMA1 mutations ends diagnostic odyssey and has prognostic implications for patients with presumed Joubert syndrome
Source: Brain Commun. 2021 Jul 16;3(3):fcab163. doi: 10.1093/braincomms/fcab163 (PMC8374969; doi:10.1093/braincomms/fcab163)
Supplement: fcab163_Supplementary_Data [file Original_submission_decision_letter.pdf › Original_submission_decision_letter.pdf]

Original Submission – Decision Letter

18-Feb-2021

Dear Prof. Sayer,

Thank you for your recent submission BRAINCOM-2021-135 entitled "Identification of LAMA1 mutations ends diagnostic odyssey and has prognostic implications for patients with presumed Joubert syndrome" to Brain Communications.

Your manuscript has been reviewed, and while the reviewers had largely positive comments, they have some constructive critiques that I think you could address in a minor revision of the manuscript. Therefore, I invite you to respond to the reviewer(s)' comments and revise your manuscript. The reviewer(s)' comments are included at the bottom of this message.

In addition to the reviewers' comments, please note our editorial comments added below to comply with our requirements for presentation of scientific data at Brain Communications.

Please format your revised manuscript according to our Instructions to Authors https://academic.oup.com/braincomms/pages/General_Instructions – a summary of key points is attached to help with this.

To revise your manuscript, click the link below or log into https://mc.manuscriptcentral.com/braincom and enter your Author Centre, where you will find your manuscript title listed under "Manuscripts with Decisions." Under "Actions," click on "Create a Revision." Your manuscript number will be appended to denote a revision.

*** PLEASE NOTE: This is a two-step process. After clicking on the link, you will be directed to a webpage to confirm. ***

https://mc.manuscriptcentral.com/braincom?URL_MASK=0f3ee9e0d2c54ace8d6059f217bd9469

Please upload a revised manuscript with the changes you have made shown either in coloured text or using "track changes".

When submitting your revised manuscript, you will be asked to respond to the reviewer(s) comments. Please be as specific as possible in your response to all of the comments.

The editorial team at Brain Communications also aims to increase transparency in scientific publishing and to demonstrate the value that peer review brings to the scientific process. Please therefore consider whether you are willing to have reviewer comments on your paper and your response published as supplementary data with the paper, if it is accepted. This is entirely optional and will only be done if both the authors

and reviewers agree. When submitting your revision you will have the opportunity to update the answer you entered previously.

IMPORTANT: Your original files are available to you when you upload your revised manuscript. Please delete any redundant files before completing the submission.

Because we are trying to facilitate timely publication of manuscripts submitted to Brain Communications, we would appreciate your revised manuscript as soon as possible. If it is not possible for you to resubmit within 3 months, the option to submit a revision will expire, and we would then consider your revised paper as a new submission. However, if you need a little more time, please get in touch and we can extend this deadline for you.

Once again, thank you for submitting your manuscript to Brain Communications and I look forward to receiving your revision.

Yours sincerely,
Dr. Jose Bras
Editor, Brain Communications
Jose.Bras@vai.org

Reviewer(s)' Comments to Author:

Reviewer: 1 (Keith Coffman)

Comments to the Author
I greatly appreciate the opportunity to review the manuscript entitled, "Identification of LAMA1 mutations ends diagnostic odyssey and has prognostic implications for patients with presumed Joubert syndrome".

The manuscript is well written, the methods are sound, the conclusions are supported by the data, and the discussion is well organized and thorough. In this manuscript, the authors present data that demonstrate four cases of misdiagnosis of Joubert Syndrome, that upon review of phenotype and further analysis of genotype, were correctly diagnosed with Poretti-Boltshauser Syndrome.

There are a number of critically important points that this manuscript brings to bear for clinicians who assess and treat patients with neurodevelopmental disorders. First, it should remind clinicians to personally review MRI images that they request on patients. The MRI images presented of the patients with confirmed Poretti-Boltshauser are clearly distinct from the example cases of Joubert Syndrome. The authors stress the role of the radiologist in recognizing the differences between the two disorders. However, thoughtful and critical review of imaging studies by the ordering clinician can also help to avoid diagnostic errors such as those demonstrated here.

Second, these data should remind clinicians that next generation sequencing analyses are typically done based on symptoms as well as phenotypic data. As the authors demonstrate, none of the patients who were discovered to have Poretti-Boltshauser syndrome had their genetic testing done with the terms "cerebellar dysplasia" or "cerebellar cysts" as part of

the phenotype. Clinicians should take heed and remember that the use of incredibly precise language is of utmost importance when pursuing NGS testing for complex patients.

Third, the data presented here underscore negative impact on patients and families that arise with incorrect diagnoses, or presumptive diagnoses without genetic confirmation. The authors state that the families had "feeling of loss and insecurity for the families involved." The importance of correct diagnosis extends past the impact on the patient and family. From a healthcare economics standpoint, getting the correct diagnoses in these cases would save significant costs as screening for cardiac, renal, and hepatic abnormalities are unnecessary in patients with Poretti-Boltshauser Syndrome.

There is one issue with the manuscript that should be addressed. On page 9, in the paragraph entitled "Exome Sequencing Data" the first sentence states, "WES was carried out for the proband and her mother". Later in the same paragraph, the authors state, "which were confirmed by Sanger sequencing and segregated from each parent". How is this possible if only mother had WES testing? Can the authors please clarify this point?

Reviewer: 2

Comments to the Author
Paper by Powel et al reports on four patients with a very characteristic clinical presentation, Poretti-Boltshauser syndrome with biallelic pathogenic variants in LAMA1. These patients were originally classified as Joubert syndrome, a genetically heterogeneous neurological ciliopathy syndrome typically associated with molar tooth sign on brain MRI. Reanalysis of the brain MRI of these four patients revealed cerebellar dysplasia and cerebellar cysts, which are typical in Poretti-Boltshauser syndrome and molar tooth sign was not detected.
This is a nice clinical genetic paper illustrating that phenotypes in children with neurogenetic conditions overlap and even the “typical” syndromes such as Joubert syndrome cannot be accurately labelled, which can delay genetic diagnosis in gene panel based approaches.
The authors also provide some prevalence data of Joubert syndrome and Poretti-Boltshauser syndrome, which has been calculated from two large UK studies (DDD, 100k genome).
1. One main message of the paper is, that if a panel based analysis do not detect a causative variant, a broader, unbiased analysis of the whole exome or genome is required in children with neurogenetic conditions. I think this should be discussed more in detail in the discussion.
2. I would be also interested in the views of the authors, whether first line genomic approach (analyzing WES, WGS) may be better suited to diagnose pediatric neurogenetic diseases in general, especially that technically in most cases WES or WGS is performed. Restricting the analysis to gene panels may miss the diagnosis in a significant number of patients. This can be illustrated will with the data reported here.
3. Figure S10 is quite informative and nice. It is demonstrating the differences between Joubert syndrome and Poretti-Boltshauser syndrome, it could be added as a figure to the main paper.

Editorial Office comments:

-Please supply a graphical abstract. A graphical abstract is a visual representation of the central finding or methodology of the paper. It will be displayed in the table of contents. Authors should strive to make the graphical abstract informative, interesting, visually appealing, and straightforward (no caption needed, no abbreviations except gene names). Please upload as a separate high resolution image file (tiff preferred). The technical requirements for the graphical abstracts are:

--size of the submitted image: 1200 pixels square at 300dpi

--font of the text: Arial 12-16 point (smaller fonts will not be legible online)

-Please add an Abbreviated Summary (up to 50 words) of your paper that captures the main purpose and conclusions of the work. The summary should state the main results written in the third person ('[1st author surname] et al. report that …') and conclusion. This summary will be used in the contents list online and should not contain any abbreviations other than those on the accepted abbreviations list and accepted gene/protein names, making it accessible to the non-specialist. Please upload it as a Word document. Allowed abbreviations: AIDS; ANOVA; AMPA; ATP; A,T,C,G; CNS; CSF; CT; DNA; ECG; EEG; EMG; GABA; HIV; MRI; NMDA; PET; PCR; RNA.

-Please include a paragraph about data availability in the 'Materials and Methods' section. We strongly encourage data sharing either as supplementary information or uploads to repositories which you can link in the paper.
