# Supplementary material for: Identification of LAMA1 mutations ends diagnostic odyssey and has prognostic implications for patients with presumed Joubert syndrome
Source: Brain Commun. 2021 Jul 16;3(3):fcab163. doi: 10.1093/braincomms/fcab163 (PMC8374969; doi:10.1093/braincomms/fcab163)
Supplement: fcab163_Supplementary_Data [file Response_to_reviewers_comments_original.pdf › Response_to_reviewers_comments_original.pdf]

Response to reviewers

Please find a point by point response to the reviewers comments, which have helped us refine and improve the manuscript.

Reviewer: 1

Comments to the Author
I greatly appreciate the opportunity to review the manuscript entitled, "Identification of LAMA1 mutations ends diagnostic odyssey and has prognostic implications for patients with presumed Joubert syndrome".
The manuscript is well written, the methods are sound, the conclusions are supported by the data, and the discussion is well organized and thorough. In this manuscript, the authors present data that demonstrate four cases of misdiagnosis of Joubert Syndrome, that upon review of phenotype and further analysis of genotype, were correctly diagnosed with Poretti-Boltshauser Syndrome.
There are a number of critically important points that this manuscript brings to bear for clinicians who assess and treat patients with neurodevelopmental disorders. First, it should remind clinicians to personally review MRI images that they request on patients. The MRI images presented of the patients with confirmed Poretti-Boltshauser are clearly distinct from the example cases of Joubert Syndrome. The authors stress the role of the radiologist in recognizing the differences between the two disorders. However, thoughtful and critical review of imaging studies by the ordering clinician can also help to avoid diagnostic errors such as those demonstrated here.
Second, these data should remind clinicians that next generation sequencing analyses are typically done based on symptoms as well as phenotypic data. As the authors demonstrate, none of the patients who were discovered to have Poretti-Boltshauser syndrome had their genetic testing done with the terms "cerebellar dysplasia" or "cerebellar cysts" as part of the phenotype. Clinicians should take heed and remember that the use of incredibly precise language is of utmost importance when pursuing NGS testing for complex patients.
Third, the data presented here underscore negative impact on patients and families that arise with incorrect diagnoses, or presumptive diagnoses without genetic confirmation. The authors state that the families had "feeling of loss and insecurity for the families involved." The importance of correct diagnosis extends past the impact on the patient and family. From a healthcare economics standpoint, getting the correct diagnoses in these cases would save significant costs as screening for cardiac, renal, and hepatic abnormalities are unnecessary in patients with Poretti-Boltshauser Syndrome.
***We wish to thank the reviewer for her/his positive appreciation of our work. The reviewer raises pertinent points (highlighted by us here in red) and we have now included or stated more explicitly some of these excellent remarks in the discussion of our manuscript.

There is one issue with the manuscript that should be addressed. On page 9, in the paragraph entitled "Exome Sequencing Data" the first sentence states, "WES was carried out for the proband and her mother". Later in the same paragraph, the authors state, "which

were confirmed by Sanger sequencing and segregated from each parent". How is this possible if only mother had WES testing? Can the authors please clarify this point?
***Thank you for highlighting this inconsistency. After exploratory WES in the proband as well as the mother (duo WES), we used DNA from both parents and the proband in order to verify the WES identified variants by Sanger sequencing as well as their segregation from each parent. This has been clarified now in the results section.

Reviewer: 2

Comments to the Author
Paper by Powel et al reports on four patients with a very characteristic clinical presentation, Poretti-Boltshauser syndrome with biallelic pathogenic variants in LAMA1. These patients were originally classified as Joubert syndrome, a genetically heterogeneous neurological ciliopathy syndrome typically associated with molar tooth sign on brain MRI. Reanalysis of the brain MRI of these four patients revealed cerebellar dysplasia and cerebellar cysts, which are typical in Poretti-Boltshauser syndrome and molar tooth sign was not detected. This is a nice clinical genetic paper illustrating that phenotypes in children with neurogenetic conditions overlap and even the “typical” syndromes such as Joubert syndrome cannot be accurately labelled, which can delay genetic diagnosis in gene panel based approaches.
The authors also provide some prevalence data of Joubert syndrome and Poretti-Boltshauser syndrome, which has been calculated from two large UK studies (DDD, 100k genome).
1. One main message of the paper is, that if a panel based analysis do not detect a causative variant, a broader, unbiased analysis of the whole exome or genome is required in children with neurogenetic conditions. I think this should be discussed more in detail in the discussion.
***We thank the reviewer for this comment. We have now added a paragraph in the discussion where we review the available evidence comparing gene panel analyses with WES in neurodevelopmental disorders. We especially highlight the limitations of targeted gene analyses and the need to extend the scope of analyses if initial targeted investigations do not detect a causative variant.
2. I would be also interested in the views of the authors, whether first line genomic approach (analyzing WES, WGS) may be better suited to diagnose pediatric neurogenetic diseases in general, especially that technically in most cases WES or WGS is performed. Restricting the analysis to gene panels may miss the diagnosis in a significant number of patients. This can be illustrated well with the data reported here.
***We thank the reviewer for this pertinent point. We have now embedded in the discussion a paragraph detailing the existing evidence in favour of an untargeted initial approach by WES. We estimate that virtual gene panels, where untargeted WES or WGS is performed, and variant interpretation then restricted to a selected number of genes have several advantages, including the possibilities of opening up the analysis to genetic phenocopies, and virtual gene panels might therefore be a reasonable first line approach in the clinical setting. In the discussion, we have highlighted the potential advantages of virtual gene panels applied to WES/WGS data in the context of the cases that we report.
3. Figure S10 is quite informative and nice. It is demonstrating the differences between Joubert syndrome and Poretti-Boltshauser syndrome, it could be added as a figure to the main paper.
***We thank the reviewer for his/her comment regarding figure S10 and have now

included it among the main figures as Figure 3 as suggested. We also will include this infographic diagram in our visual abstract to help promote the messages of the manuscript.

Editorial Office comments:

-Please supply a graphical abstract. A graphical abstract is a visual representation of the central finding or methodology of the paper. It will be displayed in the table of contents. Authors should strive to make the graphical abstract informative, interesting, visually appealing, and straightforward (no caption needed, no abbreviations except gene names). Please upload as a separate high resolution image file (tiff preferred). The technical requirements for the graphical abstracts are:
--size of the submitted image: 1200 pixels square at 300dpi
--font of the text: Arial 12-16 point (smaller fonts will not be legible online)
***We thank the editor for the suggestion to include graphical abstract which we have now provided.

-Please add an Abbreviated Summary (up to 50 words) of your paper that captures the main purpose and conclusions of the work. The summary should state the main results written in the third person (‘[1st author surname] et al. report that …’) and conclusion. This summary will be used in the contents list online and should not contain any abbreviations other than those on the accepted abbreviations list and accepted gene/protein names, making it accessible to the non-specialist. Please upload it as a Word document. Allowed abbreviations: AIDS; ANOVA; AMPA; ATP; A,T,C,G; CNS; CSF; CT; DNA; ECG; EEG; EMG; GABA; HIV; MRI; NMDA; PET; PCR; RNA.
***We have now included an abbreviated summary.

-Please include a paragraph about data availability in the 'Materials and Methods' section. We strongly encourage data sharing either as supplementary information or uploads to repositories which you can link in the paper.
***A Data Availability Statement has now been added to the Materials and Methods section
